# EEG dataset and OpenBMI toolbox for three BCI paradigms: an investigation into BCI illiteracy

**DOI:** 10.1093/gigascience/giz002

**Published:** 2019-01-30

**Authors:** Min-Ho Lee, O-Yeon Kwon, Yong-Jeong Kim, Hong-Kyung Kim, Young-Eun Lee, John Williamson, Siamac Fazli, Seong-Whan Lee

**Affiliations:** 1 Department of Brain and Cognitive Engineering, Korea University, 145 Anam-ro, Seongbuk-gu, Seoul, 02841, Korea; 2 Department of Computer Science, Nazarbayev University, Qabanbay Batyr Ave 53, Astana 010000, Kazakhstan

**Keywords:** EEG datasets, brain-computer interface, event-related potential, steady-state visually evoked potential, motor-imagery, OpenBMI toolbox, BCI illiteracy

## Abstract

**Background:**

Electroencephalography (EEG)-based brain-computer interface (BCI) systems are mainly divided into three major paradigms: motor imagery (MI), event-related potential (ERP), and steady-state visually evoked potential (SSVEP). Here, we present a BCI dataset that includes the three major BCI paradigms with a large number of subjects over multiple sessions. In addition, information about the psychological and physiological conditions of BCI users was obtained using a questionnaire, and task-unrelated parameters such as resting state, artifacts, and electromyography of both arms were also recorded. We evaluated the decoding accuracies for the individual paradigms and determined performance variations across both subjects and sessions. Furthermore, we looked for more general, severe cases of BCI illiteracy than have been previously reported in the literature.

**Results:**

Average decoding accuracies across all subjects and sessions were 71.1% (± 0.15), 96.7% (± 0.05), and 95.1% (± 0.09), and rates of BCI illiteracy were 53.7%, 11.1%, and 10.2% for MI, ERP, and SSVEP, respectively. Compared to the ERP and SSVEP paradigms, the MI paradigm exhibited large performance variations between both subjects and sessions. Furthermore, we found that 27.8% (15 out of 54) of users were universally BCI literate, i.e., they were able to proficiently perform all three paradigms. Interestingly, we found no universally illiterate BCI user, i.e., all participants were able to control at least one type of BCI system.

**Conclusions:**

Our EEG dataset can be utilized for a wide range of BCI-related research questions. All methods for the data analysis in this study are supported with fully open-source scripts that can aid in every step of BCI technology. Furthermore, our results support previous but disjointed findings on the phenomenon of BCI illiteracy.

## Data Description

### Theoretical background and purpose

A brain-computer interface (BCI) allows users to control an external device by decoding their brain activity [1]. Electroencephalography (EEG)-based BCIs have been widely used for recording brain signals because these interfaces are noninvasive, low risk, and easy to use. BCI systems have been primarily developed based on three BCI paradigms: motor imagery (MI) [2], event-related potential (ERP) [3], and steady-state visually evoked potential (SSVEP) [4]. In the past decade, BCI datasets have become freely available through BCI competitions [5], societies [6], and journal publications [7–9]. These open datasets have played an essential role in developing advanced signal processing and machine learning algorithms. Large-scale datasets have been required recently in other research fields to improve system performance, e.g., in computer vision [10,11] and brain imaging [12]. BCI systems, in particular, lacked the system performance required for real-world applications; the availability of a dataset including a large number of subjects over multiple sessions has aided in developing reliable and practical BCI systems [13,14].

Here, we present an open dataset for general-purpose BCI research. Thus, the EEG signals were recorded (1) with a large number of subjects (54 participants), (2) in multiple sessions (two sessions on different days), and (3) using multiple paradigms (MI, ERP, and SSVEP). Our dataset could, therefore, support a broad range of BCI research such as subject-dependent or independent BCI [15–17], session-to-session transfer [18], and prediction of a user’s BCI performance [19–21], among others [22]. Furthermore, we provide the BCI dataset with a laboratory developed toolbox (called “OpenBMI”) to visualize EEG data in time-frequency domains and to validate baseline performance (i.e., decoding accuracy) on the three paradigms by commonly used machine learning techniques such as common spatial pattern (CSP) [23], common spatio-spectral pattern (CSSP) [24], filter bank common spatial pattern (FBCSP) [25], Bayesian spatio-spectral filter optimization (BSSFO) [26], and canonical correlation analysis (CCA) [27].

The availability of our dataset and code offers researchers a number of advantages. First, emerging state-of-the-art techniques could be quickly evaluated using our dataset and their performance measures compared to our baseline accuracies. Second, data from our study and the open source toolbox elucidate the principles of the three major BCI systems’ architecture; therefore, our dataset is highly suitable for educational purposes in the BCI community. Third, additional research topics could be derived from our dataset as it includes various physiological signals such as EEG data for eye open/close, resting states, artifacts (e.g., head movement, eye blinking), and electromyography (EMG) data from both arms that could be suitable for machine learning and signal processing studies (e.g., optimization, artifact filtering) [28,29]. Furthermore, the dataset was recorded at a high spatial resolution (whole-head, 62 EEG electrodes) and required relatively long calibration procedures. Further neuroscientific studies on brain connectivity [30,31], neuroimaging [19], and mental workload estimation [32,33], among others, could be conducted based on our dataset.

In this study, we evaluated the inter-subject variability of BCI performance between paradigms and sessions. Additionally, the proportion of low-performance users was investigated in each paradigm individually along with the changes in that proportion between the sessions. These results are highly relevant to the study of BCI illiteracy, which affects a non-negligible portion of low-performance BCI users [34] and is a fundamental and critical issue in the current BCI literature.

Previous studies have primarily reported the problem of BCI illiteracy with respect to the MI paradigm [21,34,35] or, when examined across multiple paradigms, only with small subject groups (less than five) [36]. Anecdotal evidence suggests that MI-based BCIs suffer from a greater illiteracy rate than BCIs based on ERP [37] or SSVEP. However, to the best of our knowledge, evidence from experimental results has not been provided due to the lack of suitable datasets.

Our dataset, on the other hand, provides more conclusive evidence concerning BCI illiteracy as it includes multiple sessions and three types of BCI data from identical subjects. Firstly, we investigated the illiteracy rates in each paradigm individually along with the changes in proportion between sessions. Secondly, we categorized all subjects by their total BCI performance in the three paradigms as: (1) *universally literate*, (2) *partially literate*, or (3) *universally illiterate*.

The average rates of BCI illiteracy over the sessions were 53.7%, 11.1%, and 10.2% in the MI, ERP, and SSVEP data, respectively. These results indicate that exogenous BCI paradigms [38] (i.e., ERP and SSVEP), where external visual stimuli evoke brain responses, show a relatively small ratio of BCI illiteracy compared to the endogenous BCI paradigm [38] (i.e., MI) where a user induces the brain signals with a predefined mental task (i.e., imagined movements). Furthermore, 27.8% (15 out of 54) of users successfully performed all three BCI paradigms (universally literate), and the rest of the users were able to control at least one or two BCI paradigms (partially literate). Therefore, we reasonably conclude that general users without extraordinary handicap could use at least one of these major BCI systems.

In this study, we introduce a large-scale BCI dataset, accompanied by the OpenBMI toolbox for general-purpose BCI research. We also investigate BCI illiteracy more comprehensively in several respects with a large number of subjects over multiple sessions and paradigms. Our results provide a clearer and more general picture for the phenomenon of BCI illiteracy, which remains an important, critical issue in BCI research.

### Experimental procedure

#### Participants

Fifty-four healthy subjects (ages 24-35; 25 females) participated in the experiment. Thirty-eight subjects were naive BCI users. The others had previous experience with BCI experiments. None of the participants had a history of neurological, psychiatric, or any other pertinent disease that otherwise might have affected the experimental results. The subjects were seated comfortably in a chair with armrests at 60 (± 5) cm in front of a 21-inch LCD monitor (refresh rate: 60 Hz; resolution: 1,600 × 1,200). The approximate horizontal and vertical visual angles were 37.7 and 28.1 degrees, respectively. During the experiment, subjects were instructed to relax their muscles and minimize their eye and muscle movements.

We designed three individual BCI experiments: a binary-class MI system, a 36 symbol ERP speller, and a four target frequencies SSVEP system. All experiments followed common principles of conventional BCI research as found in [2,39,40]. All BCI experiments were developed based on the OpenBMI [41,42] and Psychophysics [43] toolboxes.

Before the experiments, subjects read instructions that provided the experimental schedule, cautions, and an explanation of the tasks. After they fully understood the experiment, questionnaire I was provided to record their personal information (e.g., age, gender) and to check their physical and mental condition. Questionnaire I included a checklist of conditions that could externally influence the subject’s BCI performance and documented their psychological and physiological state before the experiment (for details, see Table 1). Before beginning the main experiment, we recorded 10 seconds of EEG data for each of these five types of noise signals: (1) eye blinking, (2) repetitive horizontal eye movements, (3) repetitive vertical eye movements, (4) teeth clenching, and (5) flexing of both arms.

**Table 1:** Questionnaire prior to experiments

**Questionnaire I**						
Personal Information						
1	Age					
2	Gender (Male = 0, Female = 1)					
3	BCI experience (number of experiences; naive = 0)					
4	Right-handed = 0, Left-handed = 1, Ambidexter = 2					
Physiological and psychological condition						
	
1	How long have you slept?					
	(1∼4 h = 1, 5∼6 h = 2, 6∼7 h = 3, 7∼8 h = 4, >8 h = 5)					
2	Did you drink coffee in the last 24 hours?					
	(in hours since last consumption; none = 0)					
3	Did you drink alcohol in the last 24 hours?					
	(in hours since last consumption; none = 0)					
4	Did you smoke in the last 24 hours?					
	(in hours since last consumption; none = 0)					
5	Condition checklists	Low				High
	-Comfort	1	2	3	4	5
	-Motivation	1	2	3	4	5
	-Concentration	1	2	3	4	5
	-Eye fatigue	1	2	3	4	5
	-Drowsiness	1	2	3	4	5
	-Physical condition	1	2	3	4	5
	-Mental condition	1	2	3	4	5

Subjects were asked to supply their personal information and to report their physiological and psychological condition.

The main experiment consisted of ERP, MI, and SSVEP tasks in that order. The order of the paradigms was determined based on the difficulties associated with each task. The ERP-based speller system requires a relatively low mental workload compared to the MI task because the user only needs to passively gaze at the flashing target stimulus. The SSVEP paradigm is also a passive task; however, it was performed last because it is known to induce eye fatigue [44], which could influence subsequent paradigms. Each experimental task was conducted in two phases, a training phase and a test phase. In the training phase, EEG data were recorded in an offline condition and subsequently used to construct a classifier. During the test phase, real-time EEG data were acquired and decoded based on this classifier.

Our experiment required relatively long recording times, so maintaining the user’s condition and the signal quality were important. Therefore, we allowed flexible break times between experimental tasks. Impedance was checked at the end of each paradigm, and subjects were instructed to gaze at the center point of the monitor without a particular task for one minute in order to record the resting state EEG data before and after each experimental task. After each run, subjects filled out questionnaire II, which was designed with reference to [8], to check their current condition and to review the previously performed experiment (see Table 2 for details). The entire experimental procedure is summarized in Table 3.

**Table 2:** Questionnaire during the experiments

**Questionnaire II**						
Paradigm: ERP, MI or SSVEP						
Phase (offline training or online test)						
1	Are you able to participate in the following experiment?					
2	Condition check list	Low				High
	-Comfort	1	2	3	4	5
	-Motivate	1	2	3	4	5
	-Concentration	1	2	3	4	5
	-Eye fatigue	1	2	3	4	5
	-Drowsiness	1	2	3	4	5
	-Physical condition	1	2	3	4	5
	-Mental condition	1	2	3	4	5
3	Did you ever doze off or fall asleep during the experiment?					
	(number of times; none = 0)					
4	Was it easy to perform the given tasks?					
5	How many attempts have you missed?					
	(number; none = 0)					
6	Expected accuracy for this experiment (%)					

Subjects were asked to provide information regarding their current condition and self-evaluate their accuracy in the previous experiment.

**Table 3:** Experimental procedures

	Experimental procedure	Required time (min)	Cumulative time (min)
Prep. (33)	Instructions, self-assessment with questionnaire I	5	5
	EEG and EMG electrode placement	25	30
	Acquisition of artificial noise data	3	33
ERP (36)	Resting state data	1	34
	ERP speller in offline phase	12	46
	Resting state data	1	47
	Questionnaire II	2	49
	Short break	3	52
	Resting state data	1	53
	ERP speller in online phase	13	66
	Resting state data	1	67
	Questionnaire II	2	69
	Break	10	79
Motor-imagery (51)	Impedance check	5	84
	Resting state data	1	85
	Motor-imagery task in offline phase	22	107
	Resting state data	1	108
	Questionnaire II	2	110
	Short break	3	113
	Resting state data	1	114
	Motor-imagery task in online phase	22	136
	Resting state data	1	137
	Questionnaire II	2	139
	Break	10	149
SSVEP (51)	Impedance check	5	154
	Resting state data	1	155
	SSVEP task in offline phase	20	175
	Resting state data	1	176
	Questionnaire II	2	178
	Short break	3	181
	Resting state data	1	182
	SSVEP task in online phase	20	202
	Resting state data	1	203
	Questionnaire II	2	205
	Total		205

EEG data in ERP, MI, and SSVEP paradigms were sequentially recorded. Break times were flexibly adjusted with regard to the user’s condition.

#### EEG data recording

**Figure 1:**
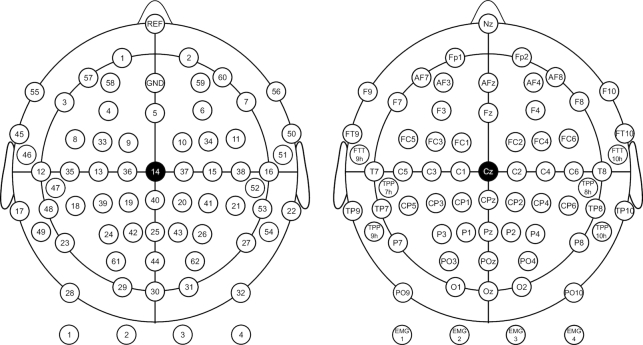
The channel configuration of the International 10-20 system (62 EEG and 4 EMG recording electrodes). The left panel indicates the indexing; the right panel corresponding location of each electrode.

EEG signals were recorded with a sampling rate of 1,000 Hz and collected with 62 Ag/AgCl electrodes. The EEG amplifier used in the experiment was a BrainAmp (Brain Products; Munich, Germany). The channels were nasion-referenced and grounded to electrode AFz. Additionally, an EMG electrode recorded from each flexor digitorum profundus muscle with the olecranon used as reference. The EEG/EMG channel configuration and indexing numbers are described in Fig. 1. The impedances of the EEG electrodes were maintained below 10 kΩ during the entire experiment.

#### ERP paradigm

**Figure 2:**
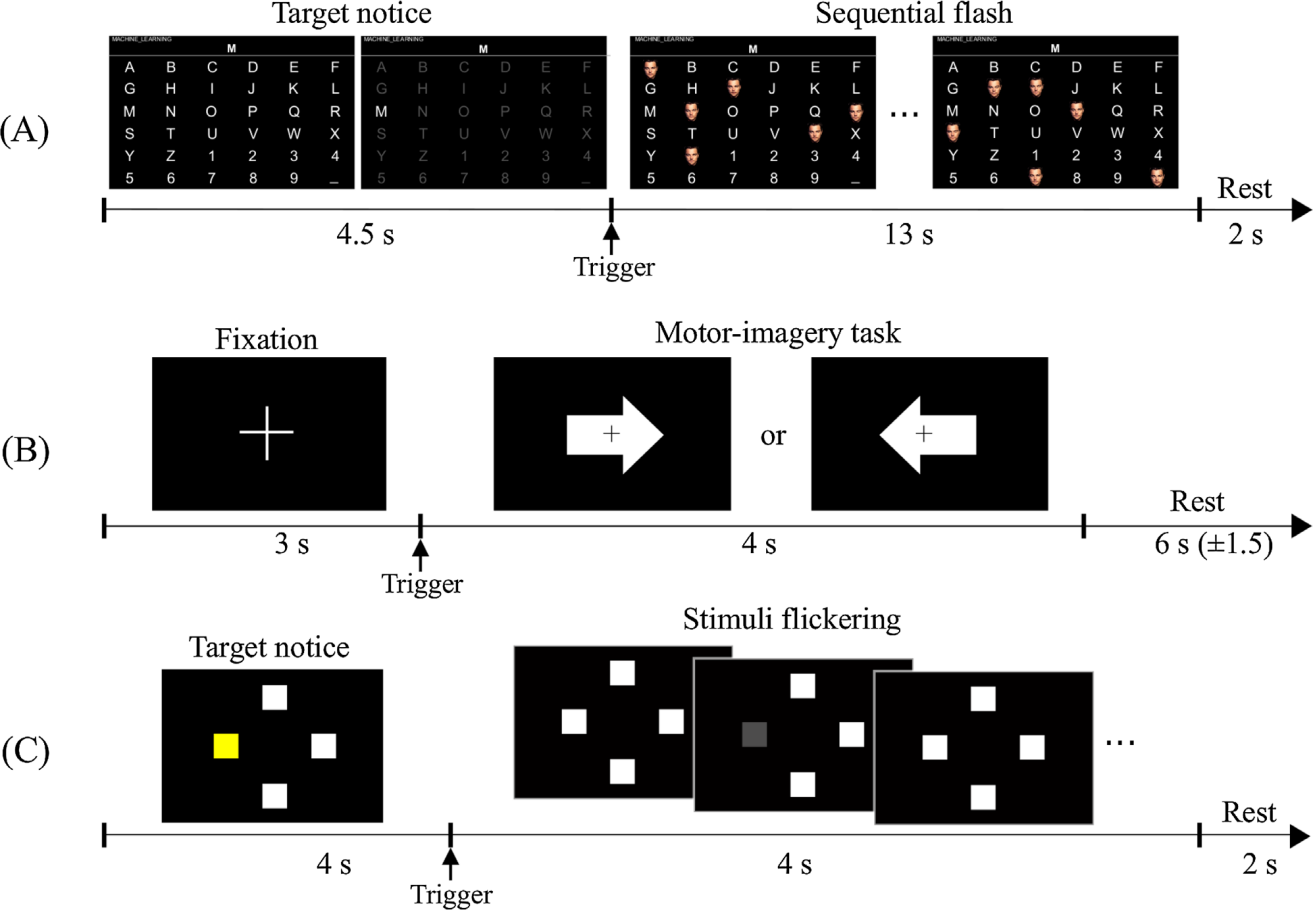
Experimental designs for the three BCI paradigms. The 6×6 ERP speller layout **(A)**, binary class MI **(B)**, and four target frequencies SSVEP **(C)** paradigms were sequentially performed.

The interface layout of the speller followed the typical design of a row-column speller. The six rows and six columns were configured with 36 symbols (A to Z, 1 to 9, and _). Each symbol was presented equally spaced (see Fig. 2A). To enhance the signal quality, two additional settings were incorporated into the original row-column speller design, namely, random-set presentation [45] and face stimuli [39]. These additional settings help to elicit stronger ERP responses by minimizing adjacency distraction errors and by presenting a familiar face image. The stimulus-time interval was set to 80 ms, and the inter-stimulus interval (ISI) to 135 ms. A single iteration of stimulus presentation in all rows and columns was considered a sequence. Therefore, one sequence consisted of 12 stimulus flashes. A maximum of five sequences (i.e., 60 flashes) was allotted without prolonged inter-sequence intervals for each target character. After the end of five sequences, 4.5 s were given to the user for identifying, locating, and gazing at the next target character. The participant was instructed to attend to the target symbol by counting the number of times each target character had been flashed.

In the training session, subjects were asked to copy-spell a given sentence, “NEURAL_NETWORKS_AND_DEEP_LEARNING” (33 characters including spaces) by gazing at the target character on the screen. The training session was performed in the offline condition, and no feedback was provided to the subject during the EEG recording. In the test session, subjects were instructed to copy-spell “PATTERN_RECOGNITION_MACHINE_LEARNING” (36 characters including spaces), and the real-time EEG data were analyzed based on the classifier that was calculated from the training session data. The selected character from the subject’s current EEG data was displayed in the top left area of the screen at the end of the presentation (i.e., after five sequences). Per participant, the collected EEG data for the ERP experiment consisted of 1,980 and 2,160 trials (samples) for training and test phase, respectively.

#### MI paradigm

The MI paradigm was designed following a well-established system protocol [2]. For all blocks, the first 3 s of each trial began with a black fixation cross that appeared at the center of the monitor to prepare subjects for the MI task. Afterwards, the subject performed the imagery task of grasping with the appropriate hand for 4 s when the right or left arrow appeared as a visual cue. After each task, the screen remained blank for 6 s (± 1.5 s). The experiment consisted of training and test phases; each phase had 100 trials with balanced right and left hand imagery tasks. During the online test phase, the fixation cross appeared at the center of the monitor and moved right or left, according to the real-time classifier output of the EEG signal (see Fig. 2B).

#### SSVEP paradigm

Four target SSVEP stimuli were designed to flicker at 5.45, 6.67, 8.57, and 12 Hz and were presented in four positions (down, right, left, and up, respectively) on a monitor. The designed paradigm followed the conventional types of SSVEP-based BCI systems that require four-direction movements [40]. Participants were asked to fixate the center of a black screen and then to gaze in the direction where the target stimulus was highlighted in a different color (see Fig. 2C). Each SSVEP stimulus was presented for 4 s with an ISI of 6 s. Each target frequency was presented 25 times. Therefore, the corrected EEG data had 100 trials (4 classes × 25 trials) in the offline training phase and another 100 trials in the online test phase. Visual feedback was presented in the test phase; the estimated target frequency was highlighted for 1 s with a red border at the end of each trial.

## Analysis

The EEG dataset was used to investigate the following areas:

- First, the detailed steps of the data analysis including offline calibration and online visual feedback have already been described. Additionally, the decoding accuracies of the three paradigms were individually validated using well-established machine learning techniques, providing a baseline accuracy.
- Second, the rate of BCI illiteracy was investigated for the individual paradigms. Furthermore, the rate of universal BCI illiteracy where the BCI user cannot control any particular BCI system was determined.
- Third, we visualized the physiological brain responses for the three BCI paradigms: event-related desynchronization/synchronization (ERD/ERS) for MI, P300 component for ERP, and band power for SSVEP paradigms.
- Fourth, the performance variations between sessions and paradigms were investigated for individual subjects.

### Data validation

The channel configurations were individually set with regard to the characteristics of each paradigm. Specifically, the MI and SSVEP paradigms highly rely on the sensory-motor and visual-cortex, respectively, so specific types of channel configuration were used in those paradigms as detailed later. A standard 32 channel montage according to International 10-20 system was selected for the ERP paradigm as the important components (e.g., P300 and N200) can be observed in broad areas of the brain. All EEG data were commonly down-sampled to 100 Hz.

For all three paradigms, our dataset is divided into a training (offline phase) and a test (online phase) dataset. The training data were used to derive classifier parameters, and the test dataset was employed for performance validation using those parameters in the MI and ERP paradigms [34]. Since the SSVEP paradigm does not require calibration data due to the characteristic of CCA analysis, the entire dataset was used for performance validation.

#### Event-related potential

For the performance validation of ERP data, 32 electrodes were selected (Fp-1/2, F-7/3/z/4/8, FC-5/1/2/6, T-7/8, C-3/z/4, TP-9/10, CP-5/1/2/6, P-7/3/z/4/8, PO-9/10, and O-1/z/2). The offline EEG data that were acquired in the training phase were band-pass filtered between 0.5 and 40 Hz with a 5th order Butterworth digital filter. The continuous EEG data were segmented from –200 to 800 ms with respect to stimulus onset and baseline-corrected by subtracting the mean amplitudes in the –200 to 0 ms pre-stimulus interval. EEG epochs in the offline phase therefore formed 100 (data points) × 32 (electrodes) × 1,980 (target and non-target trials). From the EEG epochs, subject-dependent spatio-temporal features were extracted by calculating the mean amplitudes (MA) in 10 discriminant time intervals. The linear discriminant analysis (LDA) classifier was calculated based on the feature vectors to classify the target and non-target ERP trials.

During the online test phase, the real-time data were acquired from the EEG amplifier. Preprocessing and feature extraction methods (described in a previous paragraph) were applied to the acquired EEG epoch, and the classification outputs for all individual characters were calculated using the LDA classifier constructed from the training dataset. After all five sequences, the final result for the target character was calculated by averaging the epochs from all sequences. The estimated target character was displayed on the top left area of the screen as visual feedback.

For performance validation, the classification accuracy and information transfer rates (ITRs) were calculated in each sequence (i.e., one to a maximum of five sequences). ITRs are widely used as an evaluation measure for ERP-based BCIs. The unit of ITRs is given as bits/min and can be calculated as follows:

(1) \begin{eqnarray*}
{\rm ITR} = M\left\{\log_{2} N+ {\it P}\!\log_{2} P+(1-P)\log_2\left(\frac{1-P}{N-1}\right) \right\}
\end{eqnarray*}

where *M* denotes the number of commands per minute and *N* indicates the number of possible choices, with each choice having an equal probability of being selected by the user. *P* is the accuracy of the speller (i.e., the probability that the speller selects what the user desires). In other words, the ITR corresponds to the amount of information received by the system per unit time. The gaze-shifting time for selecting a target character was not considered in the ITRs calculation.

#### Motor-imagery

For the performance validation of MI data, 20 electrodes in the motor cortex region were selected (FC-5/3/1/2/4/6, C-5/3/1/z/2/4/6, and CP-5/3/1/z/2/4/6).

The offline EEG data were band-pass filtered between 8 and 30 Hz with a 5th order Butterworth digital filter. The continuous EEG data were then segmented from 1,000 to 3,500 ms with respect to stimulus onset. EEG epochs were therefore constituted as 250 (data points) × 20 (electrodes) × 100 (trials). Frequency ranges and time intervals were selected according to previous MI studies [2,16]. CSPs were used to maximize the discrimination of the binary class [23], and log-variance features were calculated. The LDA classifier was then calculated to decode the left- or right-hand imagery task. A subset of the top and bottom two rows from the CSP projection matrix and the LDA parameters were fed to the online data analysis.

During the online test phase, a sliding window (length, 1.5 s; step size, 0.5 s) was created to classify the real-time EEG data. Specifically, the data in this window buffer were filtered with the frequency range used in the training phase, and the CSP projection matrix *w* was applied to these EEG data. The LDA outputs were calculated every 0.5 s and transformed into coordinates for the horizontal *x*-axis of the cross to provide real-time visual feedback.

The baseline performances were calculated based on well-established approaches of previous MI studies: (1) CSP [23], (2) CSSP [24], (3) FBCSP [25], and (4) BSSFO [26]. Such methods find the class-discriminative frequency bands to optimize spatial filters based on probabilistic and information-theoretic approaches. Additionally, the MI performance was validated based on 10 repetitions of 10-fold cross-validation from all MI data (i.e., training+test data) with the CSP method (CSP-cv).

#### Steady-state visually evoked potential

For the performance validation of SSVEP data, 10 electrodes in the occipital region were selected (P-7/3/z/4/8, PO-9/10, and O-1/z/2). The continuous EEG data were segmented from 0 to 4,000 ms with respect to stimulus onset. Therefore, EEG epochs were 400 (data points) × 10 (electrodes) × 100 (trials). To calculate the decoding accuracy of the four target frequency indexes, a general approach was implemented, called multi-channel CCA [27].

In CCA, a set of reference signals *Y_i_* was defined for each stimulus, including second harmonics:

(2) \begin{equation*}
Y_i(t)=\left[\begin{array}{c}
\sin(2\pi f_i t) \\
\cos(2\pi f_i t) \\
\sin(2\pi (2f_i) t) \\
\cos(2\pi (2f_i) t)
\end{array}\right], t=\frac{1}{S},\frac{2}{S},\dots,\frac{T}{S} 
\end{equation*}

where *f_i_* represents the reference frequencies (*f*_1_= 12, *f*_2_= 8.57, *f*_3_= 6.67, and *f*_4_= 5.45), *T* is the number of data points, and *S* is the sampling rate. Given a single trial *X*, the frequency index that had the highest correlation between EEG data *X* and reference signals *Y_i_* was selected as the target frequency.

### Visualization

**Figure 3:**
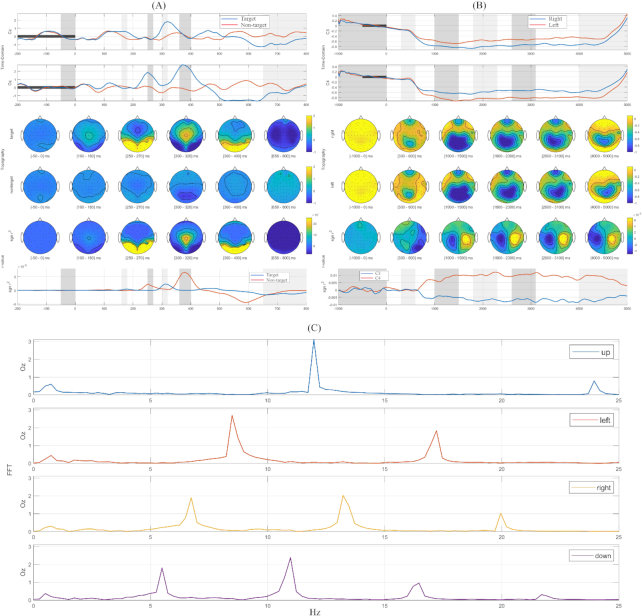
Visualization of P300 responses **(A)**, ERD/ERS patterns **(B)**, and PSD **(C)** for ERP, MI, and SSVEP data, respectively. In the visualization of ERP **(A)** and MI **(B)** data, the first two rows show grid plots in time (*x*-axis) and amplitude (*y*-axis) domains for grand-averaged brain responses in certain channels (ERP: Cz and Oz, MI: C3 and C4). The next two rows indicate the topographies of entire brain area for each class corresponding to the certain time intervals that are displayed as gray areas in the grid plot. Fifth and sixth rows present topographic and grid plot, respectively, for signed *r*-values (significance level) between the binary classes. In the visualization of SSVEP data **(C)**, one-dimensional data at Oz electrode were extracted and PSD was calculated in a frequency range of 0.1 to 25 Hz (x-axis).

Figure 3 shows grand averages of ERP, ERD/ERS, and power-spectral density (PSD) for ERP, MI, and SSVEP data, respectively. For each paradigm, the entirety of the training and test data from the two sessions and all subjects were combined.

For EEG data of the ERP paradigm, target and non-target trials were segmented in the interval of –200 ms to 800 ms with respect to stimulus onset. The Cz and Oz electrodes were representatively chosen to observe the characteristic ERP response (i.e., P300). The typical shape of ERP responses regarding the P300 component for target and non-target stimuli was visualized as reported by previous studies [9,39,45]. Positive and negative amplitudes were sufficiently represented at the central and occipital site. Specific time intervals indicated by gray areas are visualized by topographic maps as these intervals exhibit the most discriminative patterns (see Fig. 3A).

For the MI paradigm, grand-averaged ERD/ERS patterns in the mu rhythm band (8-12 Hz) are presented in Fig. 3B. The C3 and C4 electrodes, which correspond to the motor regions of the left and right hemisphere, respectively, were chosen to depict the ERD/ERS pattern induced by left- or right-hand imagery tasks. At these electrodes, the spectral power of mu rhythm significantly decreased approximately 500 ms after the stimulus onset and recovered at around end the of the task (i.e., 4,000 ms). Furthermore, antagonistic ERD patterns between contra-lateral channels were observed in the corresponding classes. Similar to the ERP plots, some intervals are emphasized by gray areas to visualize the observed changes in ERD/ERS patterns by means of topographic maps.

In the case of the SSVEP paradigm, the PSD was calculated in the frequency range of 1 to 25 Hz from SSVEP data at the Oz electrode. The PSD values were then averaged according to their class. Figure 3C indicates the PSD for the four target classes. The grid plots display significantly high amplitudes at the target frequencies corresponding to their classes. Additionally, the harmonic frequencies were also determined as described in the literature [4]. For instance, the PSD for 5.45 Hz (fourth plot in Fig. 3C) has a high amplitude at its target frequency but also at the second (10.9 Hz) and third (16.3 Hz) harmonic frequencies.

### Performance validation

The average accuracies across all 54 subjects were calculated for each of the three paradigms according to well-established approaches. Please note that our database consists of two sessions that had the same experimental protocol and subjects. Decoding accuracies in each session were calculated independently to compare their performance difference and variation. In the MI paradigm, paired *t*tests with the hypothesis of equal means were calculated between CSP and other methods (i.e., CSSP, FBCSP, and BSSFO) for each session separately.

The decoding accuracy of MI data in the first session was 70.1% (± 0.16) for CSP-cv, 67.2% (± 0.18) for CSP, 69.6% (± 0.18, *p* < 0.01) for CSSP, 68.8% (± 0.19, *p* > 0.05) for FBCSP, and 67.9% (± 0.20, *p* > 0.05) with BSSFO, and 72.2% (± 0.15), 68.5% (± 0.17, *p* > 0.05), 69.6% (± 0.18, *p* > 0.05), 70.5% (± 0.18, *p* > 0.05), and 71.1% (± 0.18, *p* > 0.05) in the second session for the respective methods.

The decoding accuracy of the ERP paradigm was calculated by averaging epochs accumulatively through the sequences (i.e., one to a maximum of five sequences). We present the decoding accuracy as well as ITRs of ERP data after five sequences. Average accuracies of ERP data were 96.5% (± 0.06) and 96.9% (± 0.05) with average ITRs of 21.1 bits/min (± 2.38) and 21.2 bits/min (± 2.10) for the first and second session, respectively.

The decoding accuracies of the SSVEP data were 94.9% (± 0.10) and 95.4% (± 0.08) in the first and second session, respectively, based on the CCA analysis.

**Figure 4:**
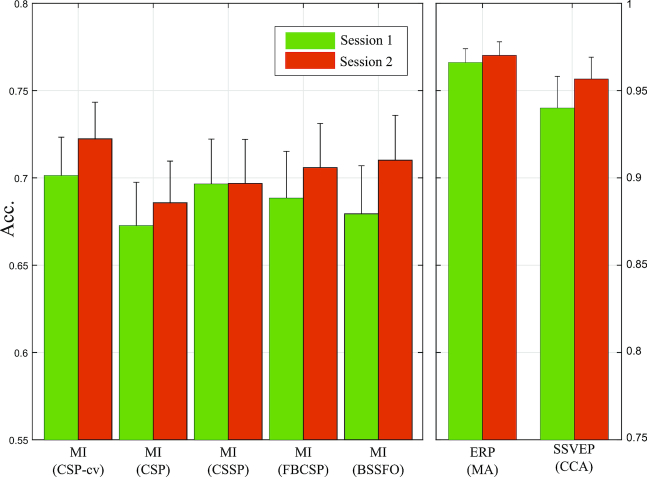
Average decoding accuracies in three BCI datasets over all subjects and sessions. The MI data were validated based on the CSP-cv, CSP, and more advanced algorithms (i.e., CSSP, FBCSP, and BSSFO). The decoding accuracies of ERP and SSVEP data were validated based on mean amplitude of ERP features and CCA, respectively.

**Figure 5:**
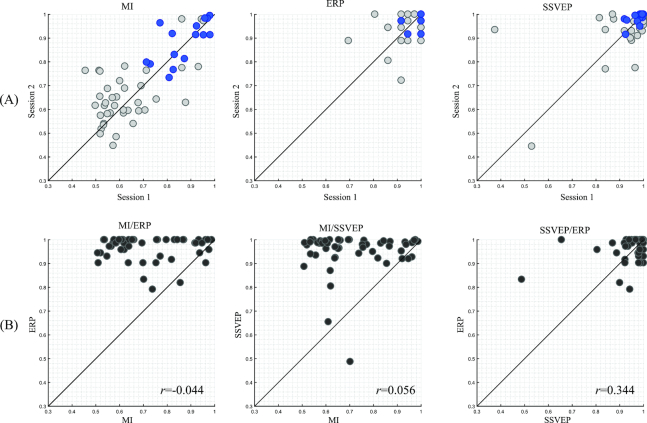
Scatter plots of performance variation across all subjects between sessions and paradigms. The first row shows variations of decoding accuracy in individual paradigms between sessions. Blue and gray circles indicate universally and partially literate BCI users, respectively, calculated in common decoding accuracy for the three BCI paradigms. The second row displays performance comparisons between paradigms (*r*, correlation coefficient).

These results indicate that the MI paradigm, in particular, exhibits large variations in decoding accuracy between subjects and sessions compared to the other paradigms (see Figs. 4 and 5A). In contrast, the SSVEP and ERP paradigms showed relatively low performance variation, and the subjects successfully performed the tasks with an average decoding accuracy of more than 90%.

Figure 5 shows scatter plots that depict the session-to-session performance variation of the individual paradigms (Fig. 5A). The mean accuracies for three paradigms in the second session were slightly higher than the first session (see Table 4). However, paired *t*tests with the hypothesis of equal means were not significant (*p* > 0.5) in all considered cases. Figure 5B illustrates scatter plots that comprise decoding accuracies of all possible paradigm pairs. After averaging the decoding accuracies in the first and second session, the correlation coefficient was calculated individually. The correlation coefficient *r* were –0.044, 0.056, and 0.344 for MI vs ERP, MI vs SSVEP, and SSVEP vs ERP, respectively. The results indicate that there is no correlation between endogenous (i.e., MI) and exogenous (i.e., ERP and SSVEP) potentials. However, a higher *r* value was observed between the two exogenous potentials ERP and SSVEP.

**Table 4:** Classification accuracy for all subjects, session and paradigms

	MI										ERP				SSVEP	
	CSP-cv		CSP		CSSP		FBCSP		BSSFO		MA-acc.		MA-ITR		CCA	
	Se1	Se2	Se1	Se2	Se1	Se2	Se1	Se2	Se1	Se2	Se1	Se2	Se1	Se2	Se1	Se2
s1^*E*^	71.3	77.2	61.0	83.0	66.0	78.0	84.0	84.0	80.0	90.0	69.4	88.9	11.8	17.9	97.5	91.0
s2	95.3	91.2	96.0	86.0	100	97.0	100	99.0	100	96.0	100	100	22.6	22.6	99.5	98.5
s3	94.7	98.3	95.0	94.0	94.0	95.0	93.0	94.0	93.0	95.0	100	100	22.6	22.6	87.5	98.0
s4^*M*^	51.4	56.5	53.0	57.0	52.0	61.0	48.0	53.0	45.0	66.0	91.7	94.4	18.9	20.0	100	100
s5^*E*^	93.3	78.8	94.0	81.0	95.0	82.0	93.0	84.0	96.0	84.0	91.7	72.2	18.9	12.6	81.5	98.5
s6	72.5	78.3	77.0	88.0	77.0	85.0	76.0	89.0	52.0	89.0	100	100	22.6	22.6	99.5	100
s7^*M*^	60.3	72.7	49.0	71.0	59.0	64.0	54.0	71.0	53.0	80.0	100	100	22.6	22.6	100	99.5
s8^*M, S*^	58.6	65.1	57.0	66.0	62.0	68.0	55.0	84.0	60.0	55.0	94.4	97.2	20.0	21.1	84.0	77.0
s9	82.1	76.4	86.0	71.0	90.0	70.0	73.0	70.0	89.0	69.0	100	100	22.6	22.6	92.0	98.0
s10^*M*^	61.8	68.8	65.0	61.0	64.0	65.0	45.0	54.0	43.0	52.0	100	100	22.6	22.6	96.0	97.0
s11^*M*^	54.7	53.6	47.0	50.0	50.0	50.0	49.0	48.0	51.0	50.0	100	100	22.6	22.6	100	100
s12^*M*^	56.1	58.1	46.0	58.0	48.0	58.0	56.0	50.0	54.0	54.0	100	97.2	22.6	21.1	100	97.0
s13^*M*^	70.0	60.5	56.0	54.0	57.0	54.0	50.0	54.0	50.0	59.0	100	100	22.6	22.6	98.5	94.0
s14^*M*^	60.9	58.4	58.0	48.0	65.0	55.0	68.0	53.0	69.0	51.0	100	100	22.6	22.6	99.5	93.0
s15^*M*^	57.9	65.1	55.0	57.0	57.0	58.0	56.0	60.0	53.0	69.0	100	100	22.6	22.6	99.5	99.5
s16^*M*^	63.8	60.5	53.0	69.0	54.0	56.0	45.0	63.0	53.0	63.0	100	100	22.6	22.6	100.0	100
s17^*M*^	80.1	75.1	83.0	42.0	90.0	45.0	88.0	54.0	81.0	55.0	94.4	91.7	20.0	18.9	98.0	98.0
s18	82.4	90.8	92.0	82.0	93.0	95.0	91.0	93.0	91.0	88.0	100	100	22.6	22.6	98.5	100
s19	83.4	83.5	82.0	89.0	85.0	83.0	89.0	89.0	83.0	82.0	100	100	22.6	22.6	98.5	98.5
s20^*M*^	51.6	76.7	59.0	73.0	53.0	79.0	50.0	82.0	52.0	62.0	100	100	22.6	22.6	99.0	95.0
s21	97.8	99.5	98.0	100	99.0	100	98.0	100	98.0	100	100	100	22.6	22.6	98.5	100
s22	86.2	78.3	77.0	85.0	91.0	92.0	92.0	65.0	94.0	90.0	94.4	88.9	20.0	17.9	94.5	90.0
s23^*M, E, S*^	63.1	78.0	54.0	68.0	51.0	57.0	55.0	55.0	58.0	53.0	86.1	80.6	16.9	15.1	53.0	44.5
s24^*M*^	54.9	57.6	49.0	54.0	48.0	66.0	50.0	45.0	51.0	51.0	100	94.4	22.6	20.0	99.0	98.5
s25^*M*^	51.7	51.2	54.0	57.0	52.0	59.0	61.0	70.0	59.0	86.0	100	88.9	22.6	17.9	100.0	95.5
s26^*M*^	59.2	46.4	49.0	44.0	58.0	44.0	52.0	48.0	45.0	48.0	86.1	94.4	16.9	20.0	98.0	99.5
s27^*M*^	52.9	62.7	56.0	70.0	55.0	62.0	47.0	55.0	44.0	51.0	100	100	22.6	22.6	99.5	99.5
s28	92.3	91.3	94.0	97.0	99.0	99.0	98.0	98.0	100	99.0	100	97.2	22.6	21.1	93.0	97.5
s29	85.5	98.0	99.0	98.0	99.0	98.0	99.0	99.0	98.0	98.0	97.2	100	21.1	22.6	95.0	89.0
s30	75.1	64.1	76.0	66.0	83.0	65.0	82.0	57.0	84.0	55.0	86.1	94.4	16.9	20.0	100	100
s31^*M*^	67.5	63.6	58.0	57.0	67.0	57.0	77.0	58.0	51.0	58.0	100	100	22.6	22.6	100	100
s32	77.3	96.1	56.0	97.0	53.0	99.0	53.0	98.0	57.0	99.0	100	100	22.6	22.6	97.5	97.0
s33	98.1	91.0	99.0	89.0	99.0	92.0	99.0	100	99.0	100	100	97.2	22.6	21.1	92.5	91.5
s34^*M, S*^	53.0	50.1	48.0	47.0	44.0	45.0	46.0	49.0	48.0	55.0	91.7	97.2	18.9	21.1	84.0	93.5
s35^*M*^	52.6	66.1	52.0	52.0	55.0	54.0	55.0	61.0	54.0	58.0	100	97.2	22.6	21.1	100	98.5
s36	96.9	98.4	97.0	94.0	99.0	94.0	98.0	98.0	98.0	100	100	91.7	22.6	18.9	100	100
s37	95.4	97.3	93.0	81.0	95.0	95.0	97.0	93.0	97.0	93.0	80.6	100	15.1	22.6	98.0	99.5
s38^*M*^	55.2	63.1	56.0	52.0	59.0	53.0	51.0	57.0	53.0	52.0	97.2	100	21.1	22.6	99.5	97.5
s39^*M*^	88.0	61.9	64.0	52.0	79.0	49.0	90.0	61.0	86.0	81.0	86.1	94.4	16.9	20.0	98.5	97.5
s40^*M*^	49.7	61.8	46.0	58.0	57.0	56.0	44.0	62.0	47.0	64.0	94.4	100	20.0	22.6	87.0	100
s41^*M*^	52.9	52.4	62.0	48.0	57.0	42.0	62.0	51.0	65.0	54.0	100	100	22.6	22.6	100	98.0
s42^*M, S*^	53.4	69.2	47.0	63.0	48.0	75.0	58.0	73.0	51.0	77.0	100	97.2	22.6	21.1	96.5	77.5
s43	86.5	81.0	77.0	86.0	90.0	90.0	87.0	89.0	91.0	95.0	100	100	22.6	22.6	99.0	100
s44	96.0	98.5	99.0	100	100	100	100	99.0	100	99.0	100	100	22.6	22.6	99.5	100
s45	92.5	95.0	93.0	99.0	94.0	99.0	95.0	98.0	93.0	100	91.7	97.2	18.9	21.1	96.5	99.5
s46^*M*^	52.7	75.7	53.0	58.0	53.0	62.0	53.0	83.0	42.0	78.0	100	94.4	22.6	20.0	92.0	93.0
s47^*M, S*^	45.2	77.3	44.0	59.0	51.0	59.0	53.0	69.0	52.0	63.0	100	100	22.6	22.6	37.5	93.5
s48^*M*^	64.2	52.7	50.0	49.0	51.0	59.0	52.0	52.0	54.0	56.0	100	100	22.6	22.6	99.0	99.5
s49^*M*^	69.6	68.8	63.0	62.0	70.0	59.0	54.0	60.0	57.0	52.0	97.2	100	21.1	22.6	100	100
s50^*M*^	61.7	60.0	59.0	58.0	59.0	55.0	58.0	48.0	58.0	50.0	91.7	100	18.9	22.6	100	97.0
s51^*M*^	68.3	58.9	71.0	52.0	65.0	48.0	59.0	52.0	62.0	49.0	91.7	88.9	18.9	17.9	94.5	90.0
s52	72.6	78.7	72.0	72.0	69.0	77.0	74.0	72.0	75.0	54.0	100	100	22.6	22.6	98.5	95.0
s53^*M*^	60.0	62.8	50.0	54.0	49.0	57.0	52.0	54.0	49.0	54.0	100	100	22.6	22.6	100	99.0
s54^*M*^	58.2	49.0	53.0	45.0	52.0	47.0	53.0	55.0	51.0	54.0	100	100	22.6	22.6	95.0	93.0
mean	70.1	72.2	67.3	68.6	69.6	69.7	68.8	70.6	67.9	71.0	96.6	97.0	21.1	21.3	94.9	95.5
std	16.2	15.4	18.3	17.6	19.0	18.5	19.8	18.6	20.3	18.8	6.2	5.4	2.4	2.1	10.9	8.6

The accuracies were validated based on CSP, CSSP, FBCSP, and BSSFO for MI, MA for ERP, and CCA for the SSVEP paradigm. The classification accuracies were validated individually for the two different sessions. Superscript symbols next to the subject number indicate illiteracy of a particular paradigm (e.g., s#^*M*^ = MI illiteracy)

**Figure 6:**
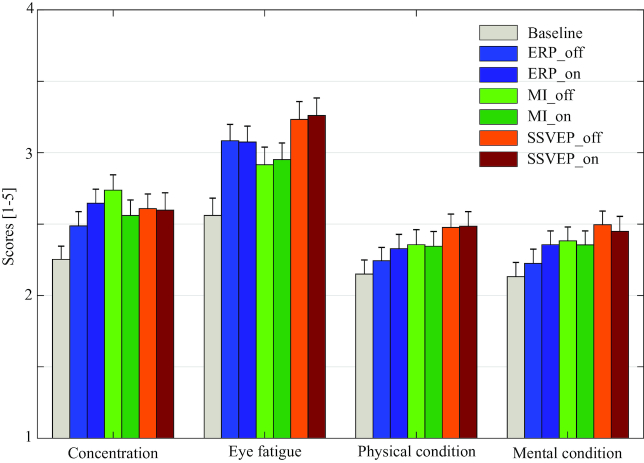
Mean rating scores of the questionnaire. Averages are calculated across all subjects and sessions. Four states such as concentration, eye-fatigue, and conditions of physical and mental state were representatively chosen (1 point: very low, 5: very high).

**Figure 7:**
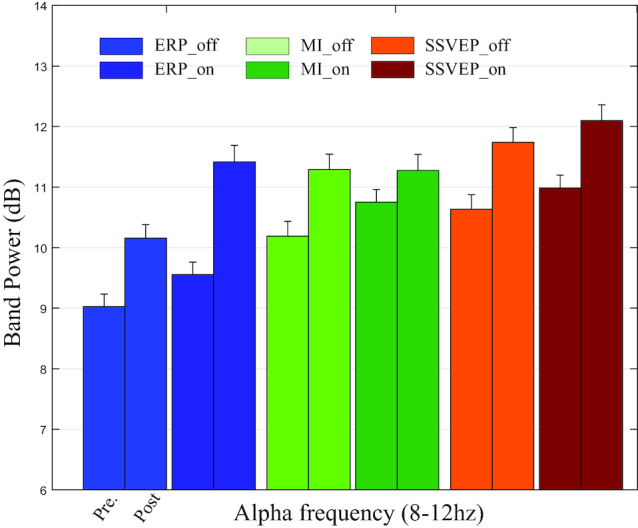
Band power (dB) of resting state data in alpha frequency range (8–12 Hz). Twenty sets of resting state date, recorded during the entire experiment, were validated (see Table 3 for further information).

Figures 6 and 7 show rating scores and band powers for the questionnaire and resting state data, respectively. For the results of the questionnaire, four states, namely, *concentration, eye-fatigue, physical condition*, and *mental condition*, were representatively selected, and reported scores for each state were averaged across the subjects and sessions. Band powers in the alpha frequency range (8–12 Hz) were calculated from resting state data and averaged across all subjects, sessions, and channels. Please refer to Table 3 for more specific information. The SSVEP and ERP paradigms showed higher eye-fatigue scores compared to the MI paradigm. This results from repetitive presentations of visual stimuli [46,47]. Average scores of subject’s physical and mental conditions and the band power in alpha frequency range were commonly increased over time. These results are in line with well-established knowledge, commonly found in the neuroscience literature, where alpha power is interpreted to be a reflection of decreased arousal levels as well as increased workloads of BCI tasks in particular [48] and other tasks in general [49,50].

### BCI illiteracy

A previous MI study defined the BCI literacy threshold at 60% to 70% [21], while the ERP and SSVEP thresholds have previously been established at 80% to 90% [35]. In accordance with these references, we set the threshold values at 70% in the MI paradigm and at 90% in the ERP and SSVEP paradigms. In the MI paradigm the mean accuracy that was used as the deciding criterion was based on the conventional CSP algorithm.

The percentages of BCI illiteracy were 55.6% (30 out of 54), 11.1% (6 out of 54), and 13.0% (7 out of 54) in the first session and 51.9% (28 out of 54), 11.1% (6 out of 54), and 7.4% (4 out of 54) in the second session for MI, ERP, and SSVEP, respectively. Additionally, we define three categories of BCI illiteracy based on their common BCI performance in the three paradigms as follows:

- **Universally literate BCI user**: a user who is able to control all three BCI paradigms.
- **Partially literate BCI user**: a user who is able to control at least one of the BCI paradigms.
- **Universally illiterate BCI user**: a user who can’t control any of the BCI paradigms.

For instance, users whose decoding accuracies for all three paradigms and sessions exceeded the predefined thresholds were attributed to the universally literate BCI group. The results indicate that 27.8% (15 out of 54) of the users can be categorized as universally literate BCI users. More importantly, we found no universally illiterate BCI user (see Fig.5A, blue and gray circles ); all subjects met at least one of the defined thresholds.

### Source code scripts

We provide fully open sourced scripts to support the data analysis of our BCI dataset. All the source codes in this study were developed based on our previous work of the OpenBMI toolbox [42]. The source codes are available on GitHub [51] and include step-by-step tutorials, which guide the user through all necessary steps of (a) recording calibration data, (b) offline analysis, and (c) on-line feedback sessions. Furthermore, the source codes includes three modules: (1) experimental protocol, (2) performance evaluation, and (3) visualization. These scripts are now freely available for all three considered paradigms and have been updated for easy use with this larger dataset. Here, we provide instructions for the toolbox with example codes so that anyone, BCI expert or beginner, can easily follow our work outlined in this paper and also implement and design new experimental paradigms of their own. Detailed documentation is also available at OpenBMI home page [41].

#### Data structure

The dataset consists of four .mat formatted files: ‘EEG_Artifact.mat’, ‘EEG_ERP.mat’, ‘EEG_MI.mat’, and ‘EEG_SSVEP.mat’. The three BCI-related .mat files contain both training and test data. For instance, the ‘EEG_MI.mat’ has two structs: ‘EEG_MI_train’ and ‘EEG_MI_test’. Individual EEG data (e.g., EEG_Artifact.mat, EEG_MI_train.mat, and etc.) are comprised of seven fields: x for continuous EEG signals (data points × channels), t for stimulus onset times of each trial, fs for sampling rates, y_dec and y_logic for class labels in integer and logical types, respectively, y_class for class definitions, and chan for channel information.

The size of the dataset is approximately 209 GB in 433 files (4 types of EEG data × 54 subjects × 2 sessions, Excel formatted data for the questionnaire, and cell_orders in the ERP paradigm). The example files (e.g., ‘Analysis_ERP.m’) in our GitHub repository describe the process of data analysis for all subjects and sessions as an aid to clearly understand each step of the analysis process.

##### Data import

Training and test MI data (*.mat format) from subject one can be loaded with the following commands:

**Figure:**
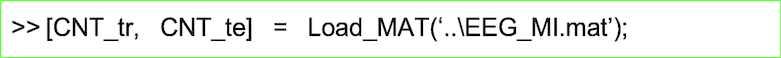


#### Data analysis

##### Preprocessing and training

The EEG data (CNT) are filtered in the frequency range of 8 to 30 Hz, and the motor-related channels are selected. The continuous EEG data are then segmented (SMT) at a predefined time interval. The spatial filter CSP_W and the classifier parameters (CF_PARAM) are calculated, and those are used to generate classifier outputs from test data.

**Figure:**
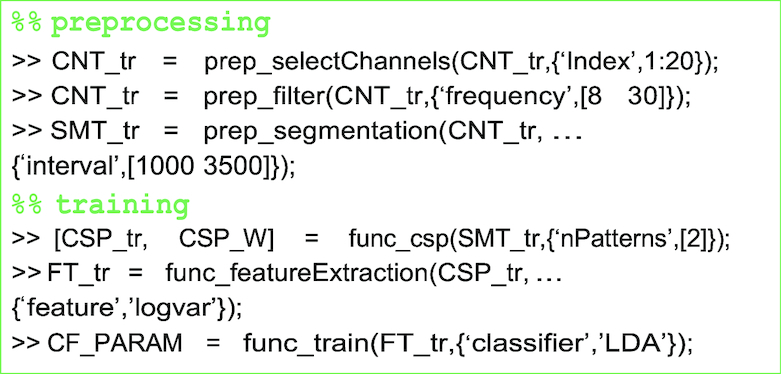


##### Performance evaluation

The test data are preprocessed with the same functions and parameters as the training data. The projection matrix CSP_W is applied to the test data and the log-variance features are extracted. The decoding accuracy is then calculated by comparison of the classifier output cf_out and true class label .y_dec of the test data.

**Figure:**
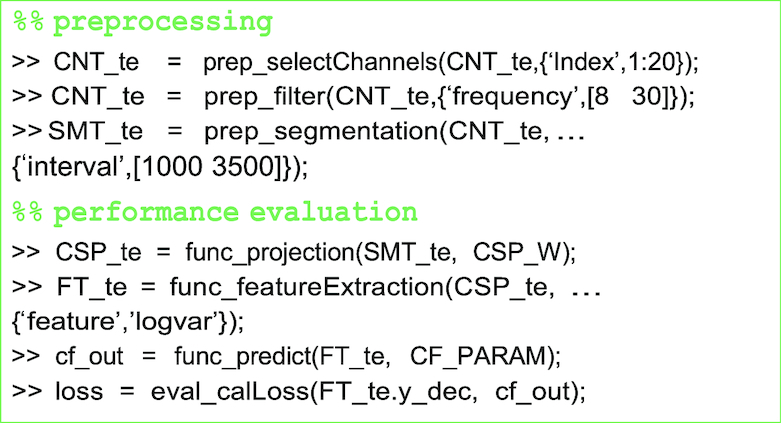


The toolbox also supports k-fold cross-validation (eval_crossValidation.m), which has been widely used for performance evaluation in MI paradigm.

#### Visualization

The GUI-based visualization module requires segmented EEG data SMT and allows easy plotting by selecting parameters such as time intervals of interest var_ival=[0 500; 1000 1500; 1500 2000; 2500 3000] and channels. Selected time intervals are highlighted in different colors on a grid plot and presented as topographic maps (see Fig. 3A and 3B).

**Figure:**
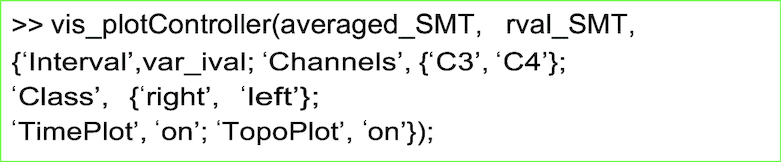


#### Experimental Protocol

Three experimental protocols are supported in offline and online conditions by the scripts Paradigm_ERP.m (ERP), Paradigm_MI.m (MI), and Paradigm_SSVEP.m (SSVEP). Users can easily modify the experimental design and the parameters according to their needs.

**Figure:**
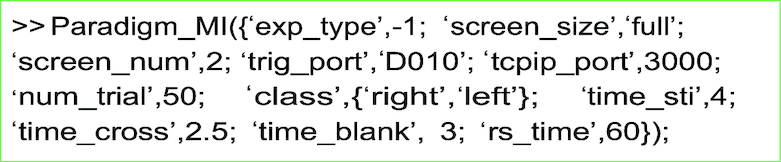


## Discussion

The classification results of all three paradigms resulted in performances that are comparable to those of previous BCI studies [8,21,35,52,53]. Specifically, the mean accuracies of MI datasets in [52] and [21] are 60.4% (± 11.6) and below 70%, respectively. Cho et al. [8] reported a mean accuracy of 67.4% (± 13.7) for 48 users. The mean accuracy of our MI data was 71.1% (± 0.15) including all 54 subjects and was 75.5% (± 0.15) when excluding 11 low-performance users (close to chance level).

The mean accuracies of ERP and SSVEP paradigms were 96.7% (± 0.15) and 95.1% (± 0.09), respectively, which were comparable in performance to previous ERP and SSVEP studies [35,53]. The decoding accuracies and the neurophysiological patterns, shown in Fig. 3, can be seen as proof of the reliability of our BCI dataset.

In this study, we investigated the BCI illiteracy rates in all three canonical paradigms. The illiteracy rates in the individual ERP, MI, and SSVEP paradigms are similar to findings in previous studies [35,52,53]. In our dataset, 27.8% of users could successfully perform all three paradigms (universally literate BCI group), and no one was deemed universally illiterate. According to these results, we conclude that most users can probably control at least one type of BCI system. Generally, we would like to note that there is some fluctuation of definition of BCI illiteracy in the literature [19,21,35]. Additionally, whether a subject is considered BCI illiterate also depends on the decoding methodology that is applied to her data.

According to Table 4, our results indicate that BCI illiteracy mostly occurs for motor imagery, which is in accordance with previous findings in the literature [9,19,20,45]. Being able to analyze a large groups of subjects who participated in the three most commonly used BCI paradigms, we were able to estimate illiteracy rates and define meaningful categories of illiteracy, such as *universally literate, partially literate*, and *universally illiterate* BCI users. Please note that, based on our criterion, none of the participants were classified as *universally illiterate* BCI users.

Concerning the users that were classified as being MI illiterate, we would like to note that in this study static band-pass filters and fixed time intervals for the estimation of CSP filters were used. From previous literature, it is known that subject-dependent estimation of these quantities can improve the decoding accuracy for motor imagery-based BCIs considerably. To date, a whole range of methods have been proposed that fulfill this task, such as heuristics [54], filter-banks [25,55], Bayesian methods [26], among many others. Here, we aimed to keep the data analytical pipeline as simple as possible and let other researchers apply their devised methodologies to the given data. As a result, we may have slightly overestimated the percentage of MI illiterates. In addition, some solutions to overcome MI illiteracy have previously been shown to be successful. Among these are multi-modal neuroimaging where, e.g., NIRS and EEG are combined [56–58], but also adaptive classification strategies have shown considerable success [34]. While it is not within the scope of this research to examine all possible routes to find solutions to this known problem, we would like to enable and invite other researches to participate in this task. We do, however, hope that our results provide more general, concrete knowledge about BCI illiteracy, which is a persistent problem for the general applicability of BCI systems.

Our dataset includes questionnaire data that contain the progression of various self-reported scores of the user’s physical and physiological conditions. Furthermore, we collected resting state data between each run. In addition, data such as EMG/EOG and artifact measurements (e.g., blinking) were also recorded. Here, we provide basic results for the questionnaire and resting state data (see Figs. 6 and 7) in order to enable other researchers to extend these findings by further analysis of individual subjects, sessions, and paradigms and by combining them with the acquired BCI data. Putting this information together facilitates investigation and proliferation of several interesting and important questions in BCI and neuroscience in general, such as mental state estimation [59,60], multi-modal data-fusion [56,61,62], and covariance shifts of the input space [63,64], among many others.

In the current BCI literature, a number of dedicated paradigm-based BCI datasets are available and can be more appropriate than our dataset for certain specialized research topics such as multi-class classification [65–68] or clinical applications [69], among others [5,6]. However, difficulties in analyzing each paradigm individually exist as those datasets have different specifications according to the recording device, experimental environment, and available toolbox. Especially for BCI studies, the procedure, system architecture, and data analysis of any given dataset are difficult to understand without a high level of background in this research field. Thus, we provide three major BCI datasets with the same specifications and with open-source scripts that fully support the entire analysis. Our dataset and the toolbox are therefore expected to increase the accessibility of BCI research for experts and beginners alike and help to easily develop typical BCI systems such as robotics [40], rehabilitation devices [70], spellers [39,45], and others.

It is our hope that this new BCI dataset and OpenBMI toolbox will be valuable to existing and new BCI researchers. With a large number of subjects, high spatial resolution, and multiple sessions across the three major paradigms, our consistent dataset provides an excellent baseline comparison, educational tool, and object of inquiry for future research in the field of BCI.

## Availability of source code and requirements

- Project name: BMIdataset
- Project home page: http://openbmi.org
- Operating system(s): Windows
- Programming language: MATLAB
- Other requirements: MATLAB 2015a or higher
- License: GPL 3.0
- Research resource identifier: OpenBMI, RRID: SCR 016876

## Availability of supporting data

The datasets and snapshots of code supporting the results of this work are available in the *GigaScience* Repository, GigaDB [71]

## Abbreviations

BCI: brain-computer interface; BSSFO: Bayesian spatio-spectral filter optimization; CCA: canonical correlation analysis; CSP: common spatial pattern; CSSP: common spatio-spectral pattern; EEG: electroencephalography; EMG: electromyography; ERD/ERS: event-related desynchronization/synchronization; ERP: event-related potential; FBCSP: filter-bank common spatial pattern; ISI: inter-stimulus interval; ITR: information transfer rate; LDA: linear discriminant analysis; MA: mean amplitude; MI: motor-imagery; PSD: power-spectral density; SSVEP: steady-state visually evoked potential.

### Ethical Approval

This study was reviewed and approved by the Korea University Institutional Review Board (1040548-KUIRB-16-159-A-2), and written informed consent was obtained from all participants before the experiments.

### Competing Interests

The authors declare that they have no competing interests.

### Author Contributions

Conceptualization, M.H.L., Y.J.K., S.W.L.; formal analysis, O.Y.K., H.K.K., Y.E.L.; data curation, O.Y.K., Y.J.K., H.K.K., Y.E.L.; software, M.H.L., O.Y.K., H.K.K.; resources, O.Y.K., Y.E.L.; investigation, M.H.L., J.W., S.F.; writing, M.H.L., J.W., S.F.; supervision, S.W.L.

## Supplementary Material

giga-d-18-00170_original_submission.pdf

giga-d-18-00170_revision_1.pdf

giga-d-18-00170_revision_2.pdf

giga-d-18-00170_revision_3.pdf

response_to_reviewer_comments_original_submission.pdf

response_to_reviewer_comments_revision_1.pdf

response_to_reviewer_comments_revision_2.pdf

reviewer_1_report_original_submission -- Xiaorong Gao 7/26/2018 Reviewed

reviewer_1_report_revision_1 -- Xiaorong Gao 11/5/2018 Reviewed

reviewer_1_report_revision_2 -- Xiaorong Gao 11/21/2018 Reviewed

reviewer_2_report_original_submission -- Dheeraj Rathee 8/5/2018 Reviewed

reviewer_2_report_revision_1 -- Dheeraj Rathee 11/6/2018 Reviewed

Supplemental Files
