# Supplementary material for: EEG dataset and OpenBMI toolbox for three BCI paradigms: an investigation into BCI illiteracy
Source: Gigascience. 2019 Jan 30;8(5):giz002. doi: 10.1093/gigascience/giz002 (PMC6501944; doi:10.1093/gigascience/giz002)

GigaScience

EEG Dataset and OpenBMI Toolbox for Three BCI Paradigms: An Investigation into BCI Illiteracy

--Manuscript Draft--

**Manuscript Number:**	GIGA-D-18-00170
**Full Title:**	EEG Dataset and OpenBMI Toolbox for Three BCI Paradigms: An Investigation into BCI Illiteracy
**Article Type:**	Research
**Funding Information:**	
**Abstract:**	Background: Electroencephalography (EEG)-based brain-computer interface (BCI) systems have mainly been divided into three major paradigms: motor-imagery (MI), event-related potential (ERP), and steady-state visually evoked potential (SSVEP). In this paper, we present a BCI dataset that includes the three major BCI paradigms with a large number of subjects over multiple sessions. In addition, information about the psychological and physiological conditions of BCI users was obtained using a questionnaire, and task-unrelated parameters such as resting state, artifacts, and electromyography of both arms were also recorded. We evaluated the decoding accuracies for the individual paradigms and determined performance variations across both, subjects and sessions. Furthermore, we looked for more general, severe cases of BCI illiteracy than have been previously reported in the literature.<br>Results: Average decoding accuracies across all subjects and sessions were 71.1% (±0.15), 96.7% (±0.05), and 95.1% (±0.09), and rates of BCI illiteracy were 53.7%, 11.1%, and 10.2% for MI, ERP, and SSVEP, respectively. Compared to the ERP and SSVEP paradigms, the MI paradigm exhibited large performance variations between both, subjects and sessions. Furthermore, we found that 27.8% (15 out of 54) of users were universally BCI literate, i.e. they were able to prociently perform all three paradigms.<br>Conclusions: Our EEG dataset can be available in a broad range of BCI study. All methods for the data analysis in this study are supported with fully open-source scripts that can aid in every step of BCI technology. Furthermore, our results support previous but disjointed ndings on the phenomenon of BCI illiteracy.
**Corresponding Author:**	Seong-Whan Lee<br>Korea University<br>Seoul, KOREA, REPUBLIC OF
**Corresponding Author Secondary Information:**	
**Corresponding Author's Institution:**	Korea University
**Corresponding Author's Secondary Institution:**	
**First Author:**	Min-He Lee
**First Author Secondary Information:**	
**Order of Authors:**	Min-He Lee
	O-Yeon Kwon
	Yong-Jeong Kim
	Hong-Kyung Kim
	Young-Eun Lee
	John Williamson
	Seong-Whan Lee
**Order of Authors Secondary Information:**	
**Additional Information:**	

Powered by Editorial Manager® and ProduXion Manager® from Aries Systems Corporation

Question	Response
Are you submitting this manuscript to a special series or article collection?	No
**Experimental design and statistics**<br><br>Full details of the experimental design and statistical methods used should be given in the Methods section, as detailed in our Minimum Standards Reporting Checklist. Information essential to interpreting the data presented should be made available in the figure legends.<br><br>Have you included all the information requested in your manuscript?	Yes
**Resources**<br><br>A description of all resources used, including antibodies, cell lines, animals and software tools, with enough information to allow them to be uniquely identified, should be included in the Methods section. Authors are strongly encouraged to cite Research Resource Identifiers (RRIDs) for antibodies, model organisms and tools, where possible.<br><br>Have you included the information requested as detailed in our Minimum Standards Reporting Checklist?	Yes
**Availability of data and materials**<br><br>All datasets and code on which the conclusions of the paper rely must be either included in your submission or deposited in publicly available repositories (where available and ethically appropriate), referencing such data using a unique identifier in the references and in the "Availability of Data and Materials" section of your manuscript.<br><br>Have you have met the above requirement as detailed in our Minimum Standards Reporting Checklist?	Yes

Powered by Editorial Manager® and ProduXion Manager® from Aries Systems Corporation

Manuscript Click here to download Manuscript Reference PDF.pdf

Placeholder for OUP logo oup.pdf

Placeholder for journal logo gigascience-logo.pdf

GigaScience, 2017, 1–13

doi: xx.xxxx/xxxx
Manuscript in Preparation
Paper

PAPER

EEG Dataset and OpenBMI Toolbox for Three BCI Paradigms: An Investigation into BCI Illiteracy

Min-Ho Lee[1], O-Yeon Kwon[1], Yong-Jeong Kim[1], Hong-Kyung Kim[1], Young-Eun Lee[1], John Williamson[1] and Seong-Whan Lee[1,*]

[1]Department of Brain and Cognitive Engineering, Korea University, 145 Anam-ro, Seongbuk-gu, Seoul, 02841, Korea

[*]Correspondence address: Department of Brain and Cognitive Engineering, Korea University, 145 Anam-ro, Seongbuk-gu, Seoul, 02841, Korea. Tel: (+82)-2-3290-3197, Fax: (+82)-2-3290-3583, E-mail: sw.lee@korea.ac.kr

Abstract

Background: Electroencephalography (EEG)-based brain-computer interface (BCI) systems have mainly been divided into three major paradigms: motor-imagery (MI), event-related potential (ERP), and steady-state visually evoked potential (SSVEP). In this paper, we present a BCI dataset that includes the three major BCI paradigms with a large number of subjects over multiple sessions. In addition, information about the psychological and physiological conditions of BCI users was obtained using a questionnaire, and task-unrelated parameters such as resting state, artifacts, and electromyography of both arms were also recorded. We evaluated the decoding accuracies for the individual paradigms and determined performance variations across both, subjects and sessions. Furthermore, we looked for more general, severe cases of BCI illiteracy than have been previously reported in the literature.
Results: Average decoding accuracies across all subjects and sessions were 71.1% ($\pm$0.15), 96.7% ($\pm$0.05), and 95.1% ($\pm$0.09), and rates of BCI illiteracy were 53.7%, 11.1%, and 10.2% for MI, ERP, and SSVEP, respectively. Compared to the ERP and SSVEP paradigms, the MI paradigm exhibited large performance variations between both, subjects and sessions. Furthermore, we found that 27.8% (15 out of 54) of users were universally BCI literate, i.e. they were able to proficiently perform all three paradigms.
Conclusions: Our EEG dataset can be available in a broad range of BCI study. All methods for the data analysis in this study are supported with fully open-source scripts that can aid in every step of BCI technology. Furthermore, our results support previous but disjointed findings on the phenomenon of BCI illiteracy.

Key words: EEG datasets; brain-computer interface; event-related potential; steady-state visually evoked potential; motor-imagery; OpenBMI toolbox; BCI illiteracy

Data Description

Theoretical background and purpose

A brain-computer interfaces (BCI) allows users to control an external device by decoding their brain activity [1]. Electroencephalography (EEG)-based BCIs have been widely used for recording brain signals because these interfaces are non-invasive, low-risk, and easy to use. BCI systems have been primarily developed based on three BCI paradigms: motor-imagery (MI) [2], event-related potential (ERP) [3], and steady-state visually evoked potential (SSVEP) [4]. In the past decade, BCI datasets have become freely available through BCI competitions [5], societies [6], and journal publications [7, 8, 9]. These open datasets have played an essential role in developing advanced signal processing and machine learning algorithms. Large-sized datasets have been required recently in other research fields to improve system performance, for example, in

Compiled on: May 11, 2018.
Draft manuscript prepared by the author.

computer vision [10, 11], and brain imaging [12]. BCI systems, in particular, lacked the system performance required for real-world application: the availability of a dataset including a large number of subjects, over multiple sessions has aided in developing reliable and practical BCI systems [13, 14].

This study presents an open dataset for general-purpose BCI research. Thus, the EEG signals were recorded with 1) a large number of subjects (54 participants), in 2) multiple sessions (two sessions on different days) and using 3) multiple paradigms (MI, ERP, and SSVEP). Our dataset could, therefore, support a broad range of BCI research such as subject-dependent or independent BCI [15, 16], session-to-session transfer [17], prediction of user's BCI performance [18], among others [19]. Furthermore, we provide the BCI dataset with a laboratory developed toolbox (called 'OpenBMI') to visualize EEG data in time-frequency domains and to validate baseline performance (i.e., decoding accuracy) on the three paradigms by commonly used machine learning techniques such as common spatial patterns (CSP) [20], common spatio-spectral pattern (CSSP) [21], filter bank common spatial pattern (FBCSP) [22], Bayesian spatio-spectral filter optimization (BSSFO) [23], and canonical correlation analysis (CCA) [24].

The availability of our dataset and code offers researchers a number of advantages. First, emerging state-of-the-art techniques could be quickly evaluated using our dataset and their performance measures compared to our baseline accuracies. Second, data from our study and the open source toolbox elucidate the principles of the 3 major BCI systems' architecture: therefore, our dataset is highly suitable for educational purposes in the BCI community. Third, additional research topics could be derived from our dataset as it includes various physiological signals such as EEG data for eye open/close, resting states, artifacts (e.g., head movement, eye blinking), and electromyography (EMG) data from both arms that could be suitable for machine learning and signal processing studies (e.g., optimization, artifact filtering) [25, 26]. Furthermore, the dataset was recorded at a high spatial resolution (whole-head, 62 EEG electrodes) and required relatively long calibration procedures; further neuroscientific studies on brain connectivity [27], neuroimaging [18], mental workload [28, 29] and others [30] could be conducted based on our dataset.

In this study, we evaluated the inter-subject variability of BCI performance between paradigms and sessions. Additionally, the proportion of low-performance users was investigated in each paradigm individually along with the changes in that proportion between the sessions. These results are highly relevant to the study of BCI illiteracy which affects a non-negligible portion of low-performance BCI users [31] and is a fundamental and critical issue in the current BCI literature.

Previous studies have primarily reported the problem of BCI illiteracy with respect to the MI paradigm [19, 31, 32] or, when examined across multiple paradigms, only with small subject groups (less than five) [33]. Anecdotal evidence suggests that MI-based BCI suffer from a greater illiteracy rate than BCI's based on ERP [34] or SSVEP. However, to the best of our knowledge, evidence from experimental results has not been provided due to the lack of suitable dataset.

Our dataset, on the other hand, provides more conclusive evidence concerning BCI illiteracy as it includes multiple session and three types of BCI data from identical subjects. Firstly, we investigated the illiteracy rates in each paradigm individually along with the changes in proportion between sessions. Secondly, we categorized all subjects by their total BCI performance in the three paradigms as either: 1) universally literate, 2) partially literate, or 3) universally illiterate.

The average rates of BCI illiteracy over the sessions were 53.7%, 11.1%, and 10.2% in the MI, ERP, and SSVEP data, respectively. These results indicate that exogenous BCI paradigms [35] (i.e., ERP and SSVEP), where external visual stimuli evoked brain signals, show a relatively small ratio of BCI illiteracy compared to the endogenous BCI paradigm [35] (i.e., MI) where a user induces the brain signals with a predefined mental task (i.e., imagined movements). Furthermore, 27.8% (15 out of 54) of users successfully performed all three BCI paradigms (universally literate), and the rest of the users were able to control one or two BCI paradigms (partially literate). Therefore, we reasonably conclude that general users without extraordinary handicap could use at least one of these major BCI systems.

In this study, we introduced our BCI dataset and OpenBMI toolbox for general purpose BCI study. We also investigated BCI illiteracy more comprehensively in several respects with a large number of subjects over multiple sessions and paradigms. Our results provided a clearer and more general picture for the phenomenon of BCI illiteracy which remains an important, critical issue in BCI research.

[Figure 1 here]

Experimental procedure

Participants

Fifty-four healthy subjects (ages 24-35, 25 females) participated in the experiment. Thirty-eight subjects were naive BCI users, and the others had at least one experience with some type of BCI experiment. None of the participants had a history of neurological, psychiatric, or any other pertinent disease that otherwise might have affected the experimental results. The subjects were seated comfortably in a chair with armrests at 60 ($\pm$5) cm in front of a 20" LCD monitor (fresh rate, 60 Hz; resolution, 1600$\times$ 1200). During the experiment, subjects were instructed to relax their muscles and minimize their eye and muscle movements.

We designed three individual BCI experiments: a binary-class MI system, a 36 symbol ERP speller, and a four target frequencies SSVEP system. All experiments followed common principles of conventional BCI research as found in [2, 36, 37]. All BCI experiments were developed based on the

Table 1. Questionnaire prior to experiments. Subjects were asked to supply their personal informations and to report their physiological and psychological condition.

Questionnaire I						
Personal Information						
1	Age					
2	Gender (Male=0, Female=1)					
3	BCI experience (number of experiences; naive=0)					
4	Right-handed=0, Left-handed=1, Ambidexter=2					
Physiological and psychological condition						
1	How long have you slept?<br>(1~4 h=1, 5~6 h=2, 6~7 h=3, 7~8 h=4, >8 h=5)					
2	Did you drink coffee in the last 24 hours?<br>(in hours since last consumption; none=0)					
3	Did you drink alcohol in the last 24 hours?<br>(in hours since last consumption; none=0)					
4	Did you smoke in the last 24 hours?<br>(in hours since last consumption; none=0)					
5	Condition check lists	Low				High
	-Comfort	1	2	3	4	5
	-Motivation	1	2	3	4	5
	-Concentration	1	2	3	4	5
	-Eye fatigue	1	2	3	4	5
	-Drowsiness	1	2	3	4	5
	-Physical condition	1	2	3	4	5
	-Mental condition	1	2	3	4	5

OpenBMI (http://openbmi.org) [38] and Psychophysics (http://psychtoolbox.org) toolboxes.

The experiment consisted of two phases, training and test phase. In the training phase, EEG data were recorded in an offline condition and were used to construct a classifier. During the test phase, real-time EEG data were acquired and decoded based on the classifier. The classification results were then presented to the user as a visual feedback. The concept of visual-neurofeedback [39] has generally been used in BCI research to acquire a high-quality EEG signal by virtue of user-adaptive training. Before the experiments, subjects read instructions that provided the experiment schedule, cautions, and an explanation of the tasks. After they fully understood the experiment, questionnaire-I was provided to record their personal information (i.e., age, gender, etc.) and to check their physical and mental condition. Questionnaire-I included a checklist for conditions that could externally influence the subject's BCI performance and documented their psychological and physiological state before the experiment (for the details, see Table 1). After each run, subjects were asked to fill out questionnaire-II to check their current condition and to review the previously performed experiment. Furthermore, subjects reported the expected accuracy in the last experiment by self-evaluating their effort on the task (see Table 2). The list in questionnaires were designed with reference to previous research [8].

Table 3 describes the entire process of the experiment. We recorded 10 seconds of EEG data for five types of noise signals; 1) eye blinking, 2) repetitive horizontal eye movements, 3) repetitive vertical eye movements, 4) teeth clenching, and 5) flexing of both arms.

The main experiment consisted of ERP, MI, and SSVEP tasks in that order. The order of the paradigms was determined based on considerations regarding their characteristic task difficulties. The ERP-based speller system requires a relatively lower mental workload compared to the MI task because the user only needs to passively gaze at the flashing target stimulus. The SSVEP paradigm is also a passive task, however, it was performed last because it induces eye-fatigue [40] which could exert an influence on subsequent paradigms. Our experiment required relatively long recording times, so maintaining the user's condition and the signal quality were important. Therefore, we allowed flexible break times between experimental tasks. Impedance was checked after the end of each paradigm. Resting state EEG data were also recorded before and after the paradigms; subjects were instructed to gaze at the center point of the monitor without a particular task for one minute.

Table 2. Questionnaire during the experiments. Subjects were asked to provide information regarding their current condition and self-evaluate their accuracy in the previous experiment.

Questionnaire II						
Paradigm: ERP, MI or SSVEP)						
Phase (offline training or online test)						
1	Are you able to participate in the following experiment?					
2	Condition check list	Low				High
	-Comfort	1	2	3	4	5
	-Motivate	1	2	3	4	5
	-Concentration	1	2	3	4	5
	-Eye fatigue	1	2	3	4	5
	-Drowsiness	1	2	3	4	5
	-Physical condition	1	2	3	4	5
	-Mental condition	1	2	3	4	5
3	Did you ever doze off or fall asleep during the experiment? (number of times; none=0)					
4	Was it easy to perform the given tasks?					
5	How many attempts have you missed? (number; none=0)					
6	Expected accuracy for this experiment (%)					

Table 3. Experimental procedures. EEG data in ERP, MI, and SSVEP paradigms were sequentially recorded. Break times were flexibly adjusted with regard to the user's condition.

	Experimental procedure	Required time (min.)	Cumulative time (min)
Prep. (33)	Instructions, self-assessment with questionnaire I	5	5
	EEG and EMG Electrode placement	25	30
	Acquisition of artificial noise data data	3	33
ERP (36)	Resting state data	1	34
	ERP speller in offline phase	12	46
	Resting state data	1	47
	Questionnaire II	2	49
	Short break	3	52
	Resting state data	1	53
	ERP speller in online phase	13	66
	Resting state data	1	67
	Questionnaire II	2	69
	Break	10	79
Motor-imagery (51)	Impedance check	5	84
	Resting state data	1	85
	Motor-imagery task in offline phase	22	107
	Resting state data	1	108
	Questionnaire II	2	110
	Short break	3	113
	Resting state data	1	114
	Motor-imagery task in online phase	22	136
	Resting state data	1	137
	Questionnaire II	2	139
	Break	10	149
SSVEP (51)	Impedance check	5	154
	Resting state data	1	155
	SSVEP task in offline phase	20	175
	Resting state data	1	176
	Questionnaire II	2	178
	Short break	3	181
	Resting state data	1	182
	SSVEP task in online phase	20	202
	Resting state data	1	203
	Questionnaire II	2	205
	Total		205

[Figure 2 here]

EEG data recording

EEG signals were recorded with a sampling rate of 1000 Hz and collected with 62 Ag/AgCl electrodes. The EEG amplifier used in the experiment was a BrainAmp (Brain Products; Munich, Germany). The channels were nasion-referenced and grounded to electrode Fpz. Additionally, an EMG electrode recorded from each flexor digitorum profundus muscle with the olecranon used as the reference. The EEG/EMG channel configuration and indexing numbers are described in Figure 1. The impedances of the EEG electrodes were maintained below 10 kΩ during the entire experiment.

ERP paradigm

The interface layout of the speller basically followed the typical design of a Row-Column speller. The six rows and six columns were configured with the 36 symbols ('A' to 'Z', '1' to '9', and '_'). Each symbol was presented equally spaced (see Figure 2-A). To enhance the signal quality, two additional settings were incorporated into the original Row-Column speller design, a random-set presentation [41] and a face stimulus [36]. These additional settings help to visualize stronger ERP responses by

minimizing adjacency distraction errors and presenting a familiar face image. The stimulus-time interval (STI) was set to 80 ms, and the inter-stimulus interval (ISI) to 135 ms. A single iteration of stimulus presentation in all rows and columns was considered a sequence. Therefore, one sequence consisted of 12 stimulus flashes, and a maximum of five sequences (i.e., 60 flashes) was allotted without prolonged inter-sequence intervals for each target character. The participant was instructed to attend to the target symbol by counting the number of times each target character had been flashed.

In the training session, subjects were asked to copy/spell a given sentence, 'NEURAL_NETWORKS_AND_DEEP_LEARNING' (33 characters including spaces) by gazing at the target character on the screen. The training session was performed in the offline condition, and no feedback was provided during the EEG recording. In the test session, subjects were instructed to copy/spell 'PATTERN_RECOGNITION_MACHINE_LEARNING' (36 characters including spaces), and the real-time EEG data were analyzed based on the classifier that was calculated in the training session. The selected character from the subject's current EEG data was displayed in the top left area of the screen at end of the presentation (i.e. after five sequences). Per participant, the collected EEG data for the ERP experiment consisted of 1980 and 2160 trials (samples) for training and test phase, respectively.

MI paradigm

The MI paradigm was designed following a well-established system protocol [2]. For all blocks, the first 3 s of each trial began with a black fixation cross that appeared at the center of the monitor to prepare subjects for the MI task. Afterwards, the subject performed the task for 4 s when the right or left arrow appeared as a visual cue. After each task, the screen remained blank for 6 s (± 1.5). The experiment consisted of training and test phases; each phase had 100 trials for balanced right and left hand imagery tasks. During the online test phase, the fixation cross appeared at the center of the monitor and moved right or left, according to the real-time classifier output of the EEG signal (see Figure 2-B).

SSVEP paradigm

Four target SSVEP stimuli were designed to flicker at 5.45, 6.67, 8.57, and 12 Hz and were presented in four positions (down, right, left, and up, respectively) on a monitor. The designed paradigm followed the conventional types of SSVEP-based BCI systems that require four-direction movements [37]. Participants were asked to fixate on the center of a black screen and then to gaze in the direction where the target stimulus was highlighted in a different color (see Figure 2-C). Each SSVEP stimulus was presented for 4 s with an ISI of 6 s. Each target frequency was presented 25 times. Therefore, the corrected EEG data had 100 trials (4 classes $\times$ 25 trials) in the offline training phase and another 100 trials in the online test phase. Visual feedback was presented in the test phase; the estimated target frequency was highlighted for one second with a red border at the end of each trial.

Analysis

The EEG dataset was used to investigate the following areas:

- First, the detailed steps of the data analysis including offline calibration and online visual feedback have already been described. Additionally, the decoding accuracies of the three paradigms were individually validated using well-established machine learning techniques, providing a baseline accuracy.
- Second, the rate of BCI illiteracy was investigated in the individual paradigms. Furthermore, the rate of universal BCI illiteracy where the BCI user cannot control any particular BCI system was also determined.
- Third, we visualized the physiological brain responses for the three BCI paradigms: event-related desynchronization/synchronization (ERD/ERS) for MI, P300 component for ERP, and band power for SSVEP paradigms.
- Fourth, the performance variations between sessions and paradigms were investigated for individual subjects.

Data validation

The channel configurations were individually set with regard to the characteristics of each paradigm. Specifically, the MI and SSVEP paradigms highly rely on the sensory-motor and visual-cortex, respectively, so specific types of channel configuration were used in those paradigms as detailed later. A standard 32 channel montage according to International 10-20 system was selected for the ERP paradigm as the important components (e.g., P300 and N200) could be observed in broad areas of the brain. All EEG data were commonly down-sampled to 100 Hz.

Our dataset is basically divided into a training (offline phase) and a test (online phase) dataset. The training data were used to derive classifier parameters, and the test data set was employed for performance validation using those parameters in the MI and SSVEP paradigms [31]. Since, the SSVEP paradigm does not require calibration data due to the characteristic of CCA analysis, the entire dataset was used in this paradigm for the performance validation.

Event-related potential

For the performance validation of ERP data, 32 electrodes were selected (Fp-1/2, F-7/3/z/4/8, FC-5/1/2/6, T-7/8, C-3/z/4, TP-9/10, CP-5/1/2/6, P-7/3/z/4/8, PO-9/10, and O-1/z/2). The offline EEG data that were acquired in the training phase were band-pass filtered between 0.5 and 40 Hz with a 5*th* order Butterworth digital filter. The continuous EEG data were segmented from -200 to 800 ms with respect to stimulus onset and baseline-corrected by subtracting the mean amplitudes in the -200 to 0 ms pre-stimulus interval. EEG epochs in the offline phase therefore formed 100 (data points)$\times$32 (electrodes)$\times$1980 (target and non-target trials). From the EEG epochs, subject-dependent spatio-temporal features were extracted by calculating the mean amplitudes (MA) in 10 discriminant time intervals. The linear discriminant analysis (LDA) classifier was calculated based on the feature vectors to classify the target and non-target ERP trials.

During the online test phase, the real-time data were acquired from the EEG amplifier. Preprocessing and feature extraction methods (described in previous paragraph) were applied to the acquired EEG epoch, and the classification outputs for all individual characters were calculated using the LDA classifier constructed from the training dataset. After all five sequences, the final result for the target character was calculated by averaging the epochs from all sequences. The estimated target character was displayed on the top left area of the screen as visual feedback.

For performance validation, the classification accuracy and information transfer rates (ITRs) were calculated in each sequence (i.e., one to a maximum of five sequences). ITRs are widely used as an evaluation measure for ERP-based BCIs. The unit of ITRs is given as bits/min and can be calculated as fol-

lows:

$$\mathrm{ITR} = M\left\{\log_2 N + P\log_2 P + (1-P)\log_2\left(\frac{1-P}{N-1}\right)\right\} \quad (1)$$

Where M denotes the number of commands per minute and N indicates the number of possible choices, with each choice having an equal probability of being selected by the user. P is the accuracy of the speller (i.e. the probability that the speller selects what the user desires). In other words, the ITR corresponds to the amount of information received by the system per unit time.

[Figure 3 here]

Motor-imagery

For the performance validation of MI data, 20 electrodes in the motor cortex region were selected (FC-5/3/1/2/4/6, C-5/3/1/z/2/4/6, and CP-5/3/1/z/2/4/6).

The offline EEG data were band-pass filtered between 8 and 30 Hz with a 5*th* order Butterworth digital filter. The continuous EEG data were then segmented from 1000 to 3500 ms with respect to stimulus onset. EEG epochs were therefore constituted as 250 (data points)×20 (electrodes)×100 (trials). Frequency ranges and time intervals were selected according to previous MI studies [2, 16]. CSPs were used to maximize the discrimination of the binary class [20], and log-variance features were calculated. The LDA classifier was then calculated to decode the left or right hand imagery task. The projection matrix from CSP and LDA parameters were fed to the online data analysis.

During the online test phase, a sliding window (length, 1.5 s; step size, 0.5 s) was created to classify the real-time EEG data. Specifically, the data in this window buffer were filtered with the frequency range used in the training phase, and the projection matrix w was applied to these EEG data. The LDA outputs were calculated every 0.5 s and transformed into coordinates for the horizontal x-axis of the cross to provide real-time visual feedback.

The baseline performances were evaluated based on general approaches in MI studies; 1) CSP [20], 2) CSSP [21], 3) FBCSP [22], and 4) BSSFO [23]. Such methods find the class-discriminative frequency bands to optimize spatial filters based on the probabilistic and information-theoretic approach. Additionally, the MI performance was validated based on ten repetitions of 10-fold cross-validation from all MI data (i.e., training+test data) with the CSP method (CSP-cv).

Steady-state visually evoked potential

For the performance validation of SSVEP data, 10 electrodes in the occipital region were selected (P-7/3/z/4/8, PO-9/10, and O-1/z/2). The continuous EEG data were segmented from 0 to 4000 ms with respect to stimulus onset. Therefore, EEG epochs were 400 (data points)×10 (electrodes)×100 (trials). To calculate the decoding accuracy of the four target frequency indexes, a general approach was implemented, called multi-channel CCA [24].

In CCA method, a set of reference signals Y_i for each stimulus included second harmonics and was defined as:

$$Y_i(t) = \begin{bmatrix} \sin(2\pi f_i t) \\ \cos(2\pi f_i t) \\ \sin(2\pi(2f_i)t) \\ \cos(2\pi(2f_i)t) \end{bmatrix}, t = \frac{1}{S}, \frac{2}{S}, \ldots, \frac{T}{S} \quad (2)$$

Where f_i represents the reference frequencies (f_1=12, f_2=8.57, f_3=6.67, and f_4=5.45), T is the number of data points, and S is the sampling rate. Given a single trial X, the frequency index that had the highest correlation between EEG data X and reference signals Y_i was selected as a target frequency.

[Figure 4 here]

Visualization

Figure 3 shows grand averages of ERP, ERD/ERS, and PSD for ERP, MI and SSVEP data, respectively. For each paradigm, the entirety of the training and test data from the two sessions and all subjects were combined.

In the case of the ERP paradigm, grand averaged target and non-target trials were in the interval of -200 ms to 800 ms with respect to stimulus onset. The Cz and Oz electrodes were representatively chosen to observe the characteristic ERP response (i.e., P300). The typical shape of ERP responses regarding the P300 component for target and non-target stimuli was visualized as reported by previous studies [9, 36, 41]. Positive and negative amplitudes were sufficiently represented at the central and occipital site. Specific time intervals indicated by gray areas, are visualized by topographic maps as these intervals exhibit the most discriminative patterns (see Figure 3-A).

In the case of the MI paradigm, the grand averaged ERD/ERS patterns in the mu rhythm band (8-12 Hz) are presented in Figure 3-B. The C3 and C4 electrodes, which correspond to the motor regions of the left and right hemisphere, respectively, were chosen to observe the ERD/ERS pattern induced by left or right hand imagery tasks. At these electrodes, the spectral power of mu rhythm significantly decreased approximately 500 ms after the stimulus onset and recovered around end of the task (i.e., 4000 ms). Furthermore, antagonistic ERD patterns between contra-lateral channels were observed in the corresponding classes. Similar to the ERP plots, some intervals are emphasized by gray areas to visualize with topographic maps the observed changes in ERD/ERS patterns at these intervals.

In the case of the SSVEP paradigm, the PSD was calculated in the frequency range of 1 to 25 Hz from SSVEP data at the Oz electrode. The PSD values were then averaged according to their class. Figure 3-C indicates the PSD for the four target classes. The grid plots display significantly high amplitudes at the target frequencies corresponding to their classes. Additionally, the harmonic frequencies were also determined as described in previous studies [4]. For instance, the PSD for 5.45 Hz (fourth plot in Figure 3-C) has a high amplitude at its target frequency, but also at the second (10.9 Hz) and third (16.3 Hz) harmonic frequencies.

[Figure 5 here]

Performance validation

The average accuracies across all 54 subjects were calculated for each of the three paradigms according to well established approaches. Please note that our database consists of two sessions that had the same experimental protocol and subjects. Decoding accuracies in each session were calculated independently to compare their performance difference and variation.

The decoding accuracy of MI data in the first session were 70.1% (±0.16) with CSP-cv, 67.2% (±0.18) with CSP, 69.6% (±0.18) with CSSP, 68.8% (±0.19) with FBCSP, and 67.9% (±0.20) with BSSFO, and 72.2% (±0.15), 68.5% (±0.17), 69.6% (±0.18), 70.5% (±0.18), and 71.1% (±0.18) in the second session for the respective methods.

The decoding accuracy of the ERP paradigm was estimated by averaging epochs accumulatively through the sequences (i.e., one to a maximum of five sequences). Here we only

present the decoding accuracy of ERP data after five sequences. Average accuracies of ERP data were 96.5% ($\pm$0.06) and 96.9% ($\pm$0.05) with average ITRs of 21.1 bits/min ($\pm$2.38) and 21.2 bits/min ($\pm$2.10) for the first and second session, respectively.

The decoding accuracies of the SSVEP data were 94.9% ($\pm$0.10) and 95.4% ($\pm$0.08) in the first and second session, respectively, based on the CCA analysis.

The results indicate that the MI paradigm, in particular, exhibits large variations in decoding accuracy between subjects and sessions compared to the other paradigms (see Figure 4 and Figure 5-A). In contrast, the SSVEP and ERP paradigms showed relatively low performance variation, and the subjects successfully performed the tasks with an average decoding accuracy at the 90% level.

Figure 5 shows scatter plots that depict the session-to-session performance variation in individual paradigms (Figure 5-A). Figure 5-B illustrates the comparison of decoding accuracies in pair sets of the paradigm. After averaging the decoding accuracies in the first and second session, the correlation coefficient was calculated individually. The correlation coefficient r were -0.044, 0.056, and 0.344 for MI vs. ERP, MI vs. SSVEP, and SSVEP vs. ERP, respectively. The results indicate that there is no correlation between endogenous (i.e., MI) and exogenous (i.e., ERP and SSVEP) potentials. However, a relatively higher r value was observed between the two exogenous potentials ERP and SSVEP.

BCI illiteracy

To investigate the rate of BCI illiteracy, decoding accuracies of session one and two were averaged. A previous MI study defined the BCI literacy threshold at 60 to 70% [19], while the ERP and SSVEP threshold was established at 80 to 90% [32]. In accordance with these references, we set the threshold values at 70% in the MI paradigm and at 90% in the ERP and SSVEP paradigms.

The percentages of BCI illiteracy were 55.6% (30 out of 54), 11.1% (6 out of 54), and 13.0% (7 out of 54) in the first session and 51.9% (28 out of 54), 11.1% (6 out of 54), and 7.4% (4 out of 54) in the second session for MI, ERP, and SSVEP, respectively. Additionally, we define three categories of BCI illiteracy based on their common BCI performance in the three paradigms as follows:

- **Universally literate BCI user**: a user who is able to control all three BCI paradigms.
- **Partially literate BCI user**: a user who is able to control at least one of the BCI paradigms.
- **Universally illiterate BCI user**: a user who can't control any of the BCI paradigms.

For instance, users whose decoding accuracies for all three paradigms and sessions exceeded the predefined thresholds were attributed to the universally literate BCI group. The results indicate that 27.8% (15 out of 54) of the users were categorized as universally literate BCI users. More importantly, we found no universally illiterate BCI user (see 5-first row, blue and gray circles); all subjects met at least one of the defined thresholds.

OpenBMI toolbox

We provide an open-source toolbox to support the data analysis of our BCI dataset. All the source code in this study was developed based on our previous work of the OpenBMI toolbox [38]. The OpenBMI toolbox covers all the steps of data analysis that were used in this paper. The scripts include three modules: 1) experimental protocol, 2) performance evaluation, and 3) visualization. Here, we provide instructions for the toolbox with example code so that anyone, BCI expert or beginner, can easily follow our work. Detailed documentation is also available at https://openbmi.org.

Data structure

EEG data comprises seven fields: `x` for continuous EEG signals (data points $\times$ channels), `t` for stimulus onset times of each trial, `fs` for sampling rates, `y_dec` and `y_logic` for class labels in number and logical types, respectively, `y_class` for class definition, and `chan` for channel information.

Data import. Training and test MI data (`*.mat` format) from subject one can be loaded with the following commands:

```
>> [CNT_tr, CNT_te] = Load_MAT('..\EEG_MI.mat');
```

Data analysis

Preprocessing and training. The EEG data (`CNT`) are filtered in the frequency range of 8 to 30 hz, and the motor-related channels are selected. The continuous EEG data are then segmented (`SMT`) at a predefined time interval. The spatial filter `CSP_W` and the classifier parameters (`CF_PARAM`) are calculated, and those are used to generate classifier outputs from test data.

```
%% preprocessing
>> CNT_tr = prep_selectChannels(CNT_tr,{'Index',1:20});
>> CNT_tr = prep_filter(CNT_tr,{'frequency',[8 30]});
>> SMT_tr = prep_segmentation(CNT_tr, ...
{'interval',[1000 3500]});
%% training
>> [CSP_tr, CSP_W] = func_csp(SMT_tr,{'nPatterns',[2]});
>> FT_tr = func_featureExtraction(CSP_tr, ...
{'feature','logvar'});
>> CF_PARAM = func_train(FT_tr,{'classifier','LDA'});
```

Performance evaluation. The test data are preprocessed with the same conditions applied as in the training steps. The projection matrix w is applied to the test data and the log-variance features are extracted. The decoding accuracy is then calculated by comparison of the classifier output `cf_out` and true class label `.y_dec` of the test data.

```
%% preprocessing
>> CNT_te = prep_selectChannels(CNT_te,{'Index',1:20});
>> CNT_te = prep_filter(CNT_te,{'frequency',[8 30]});
>> SMT_te = prep_segmentation(CNT_te, ...
{'interval', [1000 3500]});
%% performance evaluation
>> CSP_te = func_projection(SMT_te, CSP_W);
>> FT_te = func_featureExtraction(CSP_te, ...
{'feature','logvar'});
>> cf_out = func_predict(FT_te, CF_PARAM);
>> loss = eval_calLoss(FT_te.y_dec, cf_out);
```

The toolbox also supports k-fold cross-validation (`eval_crossValidation.m`), which has widely used for performance evaluation in MI paradigm.

Visualization

The GUI-based visualization module requires segmented EEG data `SMT`, and allows easy plotting by selecting some parameters such as time intervals of interest `var_ival=[0 500; 1000 1500; 1500 2000; 2500 3000]` and channels. Selected time intervals are highlighted in different

colors on grid plot and presented on the topographic maps (see Figure 3-A and B).

```
>> vis_plotController(averaged_SMT, rval_SMT,
{’Interval’,var_ival; ’Channels’, {C3, C4};
’Class’, {’right’, ’left’};
’TimePlot’, ’on’; ’TopoPlot’, ’on’);
```

Experimental Protocol

Three experimental protocols are supported in offline and online conditions by the scripts `Paradigm_ERP.m` (ERP), `Paradigm_MI.m` (MI), and `Paradigm_SSVEP.m` (SSVEP). Users can easily modify the experimental design and the parameters according to their needs.

```
>> Paradigm_MI({’exp_type’,-1; ’screen_size’,’full’;
’screen_num’,2; ’trig_port’,’D010’; ’tcpip_port’,3000;
’num_trial’,50; ’class’,{’right’,’left’}; ’time_sti’,4;
’time_cross’,2.5; ’time_blank’, 3; ’rs_time’,60});
```

Discussion

The classification results in all three paradigms achieved performances comparable to those in previous BCI studies [8, 19, 32, 42, 43]. Specifically, the mean accuracies of MI datasets in [42] and [19] are 60.4% (±11.6) and below 70%, respectively. In [8] reported the mean accuracy of 67.4% (±13.7) for 48 users. The mean accuracy of our MI data was 71.1% (±0.15) including all 54 subjects and was 75.5% (±0.15) when excluding 11 low performance users (close to random chance level).

The mean accuracies of ERP and SSVEP paradigms were 96.7% (±0.15) and 95.1% (±0.09), respectively, which were comparable in performance to previous ERP and SSVEP studies [32, 43]. The decoding accuracies and the neurophysiological patterns, showed in Figure 3, were also proof of the reliability of our BCI dataset.

In this study, we investigated the BCI illiteracy rates in all three canonical paradigms. The illiteracy rates in the individual ERP, MI, and SSVEP paradigms are similar to findings in previous studies [32, 42, 43]. In our dataset, 27.8% of users could successfully perform all three paradigms (universally literate BCI group), and no one was deemed universally illiterate. According to these results, we conclude that most users can probably control at least one type of BCI system.

We hope that our results provide more general, concrete knowledge about BCI illiteracy which is a persistent problem for the general applicability of BCI systems.

In the current BCI literature, a number of dedicated paradigm-based BCI datasets are available. However, difficulties in analyzing each paradigm individually exist as those datasets have different specifications according to the recording device, experimental environment, and available toolbox. Especially for BCI studies, the procedure, system architecture, and data analysis of any given dataset are difficult to understand without a high level of background in this research field. Thus, we provide three major BCI datasets with the same specifications and with open-source scripts which fully support the entire BCI system. Our dataset and the toolbox are therefore expected to increase the accessibility of BCI research for experts and beginners alike and help to easily develop typical BCI systems such as robotics [37], rehabilitation devices [39], spellers [36, 41], and others.

It is our hope that this new BCI dataset and OpenBMI toolbox will be valuable to existing and new BCI researchers. With a large number of subjects, high spatial resolution, and multiple sessions across the three major paradigms, our consistent dataset provides an excellent baseline comparison, educational tool, and object of inquiry for future research in the field of BCI.

Availability of source code and requirements

Lists the following:

- Project name: OpenBMI
- Project home page: http://openbmi.org
- Operating system(s): Windows
- Programming language: MATLAB
- Other requirements: MATLAB 2015a or higher
- License: GPL 3.0 Any restrictions to use by non-academics: licence needed

Declarations

List of abbreviations

BCI: brain-computer interface; BSSFO: Bayesian spatio-spectral filter optimization; CCA: canonical correlation analysis ; CSP: common spatial pattern; CSSP: common saptio-spectral pattern; CV: cross-validation; EEG: electroencephalography; EMG: electromyography; ERD/ERS: event-related desynchronization/synchronization; ERP: event-related potential; FBCSP: filter-bank common spatial pattern; ISI: inter-stimulus interval; ITRs: information transfer rates; LDA: linear discriminant analysis; MA: mean amplitude; MI: motor-imagery; PSD: power-spectral density; SSVEP: steady-state visually evoked potential; STI: stimulus-time interval.

Ethical Approval

This study was reviewed and approved by the Institutional Review Board at Korea University [1040548-KUIRB-16-159-A-2], and written informed consent was obtained from all participants before the experiments.

Competing Interests

The authors declare that they have no competing interests.

Author’s Contributions

MHL, OYK, and HKK developed the open source toolbox and validated performance of the dataset. OYK, YJK, and YEL collected the dataset. MHL, JW, and SWL wrote the draft. SWL supervised the entire project.

Acknowledgment

This research was supported by the MSIT (Ministry of Science and ICT), Korea, under the SW Starlab support program (IITP-2015-1107) supervised by the IITP (Institute for Information & Communications Technology Promotion) and funded by the Korea government (No. 2017-0-00451, Development of BCI based Brain and Cognitive Computing Technology for Recognizing User’s Intentions using Deep Learning).

References

1. Wolpaw JR, Birbaumer N, Heetderks WJ, McFarland DJ, Peckham PH, Schalk G, et al. Brain-computer interface technology: a review of the first international meeting. IEEE Transactions on Rehabilitation Engineering 2000;8(2):164–173.
2. Pfurtscheller G, Neuper C. Motor imagery and direct brain-computer communication. Proceedings of the IEEE 2001;89(7):1123–1134.
3. Picton TW. The P300 wave of the human event-related potential. Journal of Clinical Neurophysiology 1992;9(4):456–479.
4. Müller-Putz GR, Scherer R, Brauneis C, Pfurtscheller G. Steady-state visual evoked potential (SSVEP)-based communication: impact of harmonic frequency components. Journal of Neural Engineering 2005;2(4):123.
5. BBCI competition Datasets;. Accessed: 2018-04-09. http://www.bbci.de/competition.
6. BNCI Horizon 2020 Datasets;. Accessed: 2018-04-09. http://bnci-horizon-2020.eu/database/data-sets.
7. Wang Y, Chen X, Gao X, Gao S. A benchmark dataset for SSVEP-based brain-computer interfaces. IEEE Transactions on Neural Systems and Rehabilitation Engineering 2017;25(10):1746–1752.
8. Cho H, Ahn M, Ahn S, Kwon M, Jun SC. EEG datasets for motor imagery brain computer interface. GigaScience 2017;.
9. Vareka L, Bruha P, Moucek R. Event-related potential datasets based on a three-stimulus paradigm. GigaScience 2014;3(1):35.
10. Krizhevsky A, Sutskever I, Hinton GE. Imagenet classification with deep convolutional neural networks. In: Advances in Neural Information Processing Systems; 2012. p. 1097–1105.
11. The MNIST database;. Accessed: 2018-04-09. http://yann.lecun.com/exdb/mnist.
12. Open-Access Medical Image Repositories;. Accessed: 2018-04-09. http://www.aylward.org/notes/open-access-medical-image-repositories.
13. Hosseini MP, Pompili D, Elisevich K, Soltanian-Zadeh H. Optimized deep learning for EEG big data and seizure prediction BCI via internet of things. IEEE Transactions on Big Data 2017;3(4):392–404.
14. Friedenberg DA, Bouton CE, Annetta NV, Skomrock N, Zhang M, Schwemmer M, et al. Big data challenges in decoding cortical activity in a human with quadriplegia to inform a brain computer interface. In: Engineering in Medicine and Biology Society (EMBC), 2016 IEEE 38th Annual International Conference of the IEEE; 2016. p. 3084–3087.
15. Guger C, Ramoser H, Pfurtscheller G. Real-time EEG analysis with subject-specific spatial patterns for a brain-computer interface (BCI). IEEE Transactions on Rehabilitation Engineering 2000;8(4):447–456.
16. Fazli S, Popescu F, Danóczy M, Blankertz B, Müller KR, Grozea C. Subject-independent mental state classification in single trials. Neural Networks 2009;22(9):1305–1312.
17. Samek W, Meinecke FC, Müller KR. Transferring subspaces between subjects in brain-computer interfacing. IEEE Transactions on Biomedical Engineering 2013;60(8):2289–2298.
18. Blankertz B, Sannelli C, Halder S, Hammer EM, Kübler A, Müller KR, et al. Neurophysiological predictor of SMR-based BCI performance. Neuroimage 2010;51(4):1303–1309.
19. Ahn M, Cho H, Ahn S, Jun SC. High theta and low alpha powers may be indicative of BCI-illiteracy in motor imagery. PLOS ONE 2013;8(11):e80886.
20. Ramoser H, Muller-Gerking J, Pfurtscheller G. Optimal spatial filtering of single trial EEG during imagined hand movement. IEEE Transactions on Rehabilitation Engineering 2000;8(4):441–446.
21. Lemm S, Blankertz B, Curio G, Muller KR. Spatio-spectral filters for improving the classification of single trial EEG. IEEE Transactions on Biomedical Engineering 2005;52(9):1541–1548.
22. Ang KK, Chin ZY, Zhang H, Guan C. Filter bank common spatial pattern (FBCSP) in brain-computer interface. In: Neural Networks, 2008. IJCNN 2008.(IEEE World Congress on Computational Intelligence). IEEE International Joint Conference on IEEE; 2008. p. 2390–2397.
23. Suk HI, Lee SW. A novel Bayesian framework for discriminative feature extraction in brain-computer interfaces. IEEE Transactions on Pattern Analysis and Machine Intelligence 2013;35(2):286–299.
24. Lin Z, Zhang C, Wu W, Gao X. Frequency recognition based on canonical correlation analysis for SSVEP-based BCIs. IEEE Transactions on Biomedical Engineering 2007;54(6):1172–1176.
25. LeVan P, Urrestarazu E, Gotman J. A system for automatic artifact removal in ictal scalp EEG based on independent component analysis and Bayesian classification. Clinical Neurophysiology 2006;117(4):912–927.
26. Fatourechi M, Bashashati A, Ward RK, Birch GE. EMG and EOG artifacts in brain computer interface systems: A survey. Clinical Neurophysiology 2007;118(3):480–494.
27. Hamedi M, Salleh SH, Noor AM. Electroencephalographic motor imagery brain connectivity analysis for BCI: a review. Neural Computation 2016;28(6):999–1041.
28. Käthner I, Wriessnegger SC, Müller-Putz GR, Kübler A, Halder S. Effects of mental workload and fatigue on the P300, alpha and theta band power during operation of an ERP (P300) brain-computer interface. Biological Psychology 2014;102:118–129.
29. Roy RN, Bonnet S, Charbonnier S, Campagne A. Mental fatigue and working memory load estimation: interaction and implications for EEG-based passive BCI. In: Engineering in Medicine and Biology Society (EMBC), 2013 35th Annual International Conference IEEE; 2013. p. 6607–6610.
30. Sakkalis V. Review of advanced techniques for the estimation of brain connectivity measured with EEG/MEG. Computers in Biology and Medicine 2011;41(12):1110–1117.
31. Vidaurre C, Blankertz B. Towards a cure for BCI illiteracy. Brain Topography 2010;23(2):194–198.
32. Allison B, Luth T, Valbuena D, Teymourian A, Volosyak I, Graser A. BCI demographics: How many (and what kinds of) people can use an SSVEP BCI? IEEE Transactions on Neural Systems and Rehabilitation Engineering 2010;18(2):107–116.
33. Karat J, Vanderdonckt J. Human-Computer Interaction Series;.
34. Conroy MA, Polich J. Normative variation of P3a and P3b from a large sample: Gender, topography, and response time. Journal of Psychophysiology 2007;21(1):22.
35. Nicolas-Alonso LF, Gomez-Gil J. Brain computer interfaces, a review. Sensors 2012;12(2):1211–1279.
36. Kaufmann T, Schulz S, Grünzinger C, Kübler A. Flashing characters with famous faces improves ERP-based brain-computer interface performance. Journal of Neural Engineering 2011;8(5):056016.
37. Parini S, Maggi L, Turconi AC, Andreoni G. A robust and self-paced BCI system based on a four class SSVEP paradigm: algorithms and protocols for a high-transfer-rate direct brain communication. Computational Intelligence and Neuroscience 2009;2009.

38. Lee MH, Fazli S, Kim KT, Lee SW. Development of an open source platform for brain-machine interface: OpenBMI. In: 2016 4th International Winter Conference on Brain-Computer Interface (BCI) IEEE; 2016. p. 1–2.
39. Hwang HJ, Kwon K, Im CH. Neurofeedback-based motor imagery training for brain–computer interface (BCI). Journal of Neuroscience Methods 2009;179(1):150–156.
40. Csathó Á, Van Der Linden D, Hernádi I, Buzás P, Kalmár G. Effects of mental fatigue on the capacity limits of visual attention. Journal of Cognitive Psychology 2012;24(5):511–524.
41. Yeom SK, Fazli S, Müller KR, Lee SW. An efficient ERP-based brain-computer interface using random set presentation and face familiarity. PLOS ONE 2014;9(11):e111157.
42. Guger C, Edlinger G, Harkam W, Niedermayer I, Pfurtscheller G. How many people are able to operate an EEG-based brain-computer interface (BCI)? IEEE Transactions on Neural Systems and Rehabilitation Engineering 2003;11(2):145–147.
43. Guger C, Daban S, Sellers E, Holzner C, Krausz G, Carabalona R, et al. How many people are able to control a P300-based brain–computer interface (BCI)? Neuroscience Letters 2009;462(1):94–98.

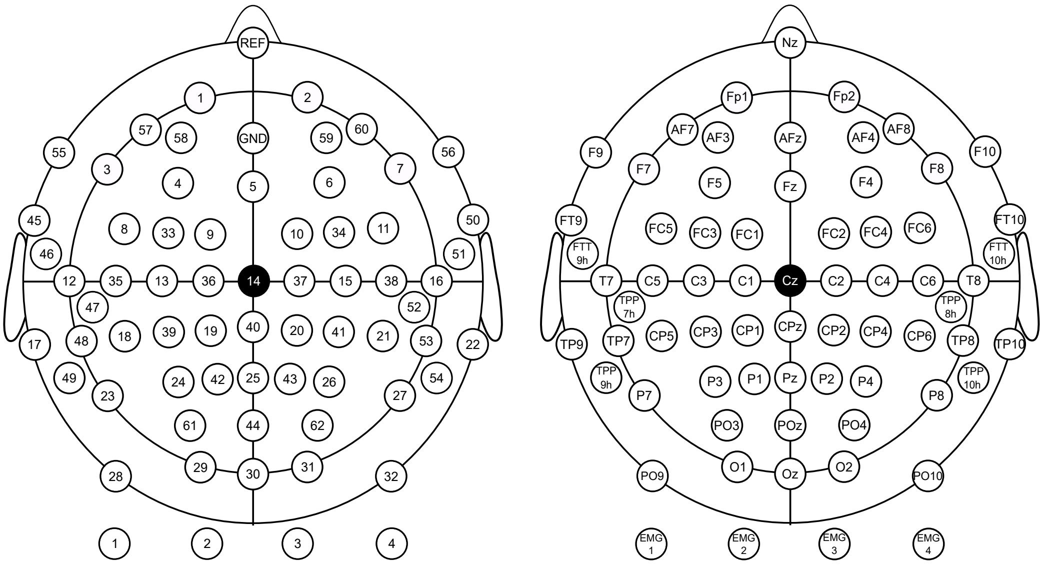

Figure 1. The channel configuration of the International 10-20 system (62 EEG and 4 EMG recording electrodes). The left panel indicates the indexing, the right panel corresponding location of each electrode.

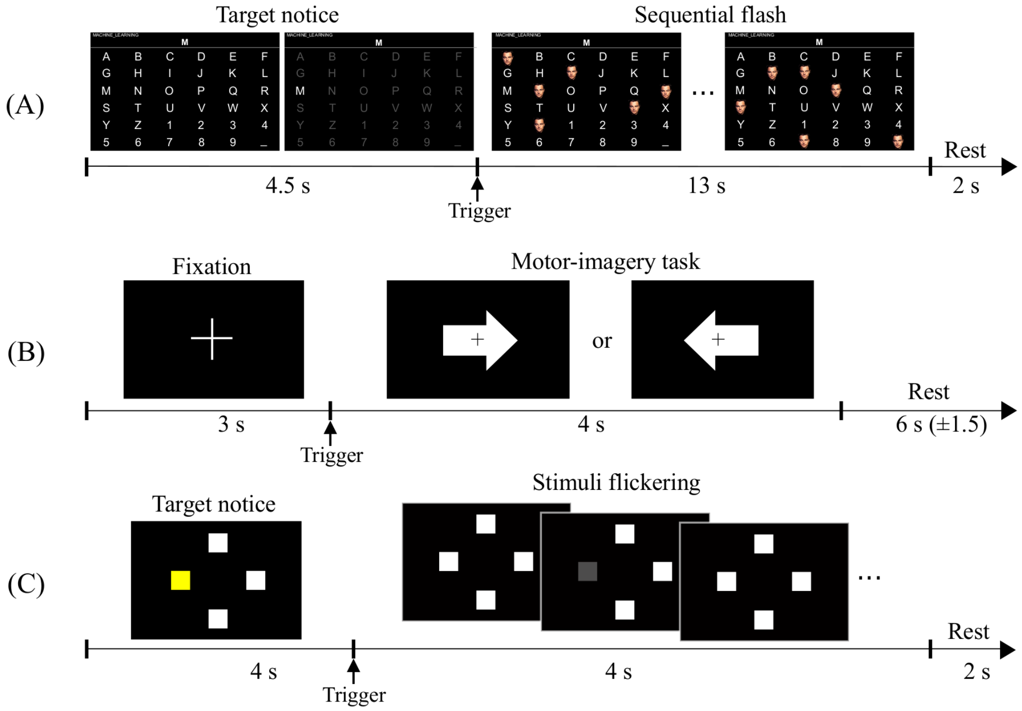

Figure 2. Experimental designs for the three BCI paradigms. The 6×6 ERP speller layout (A), binary class MI (B), and four target frequencies SSVEP (C) paradigms were sequentially performed.

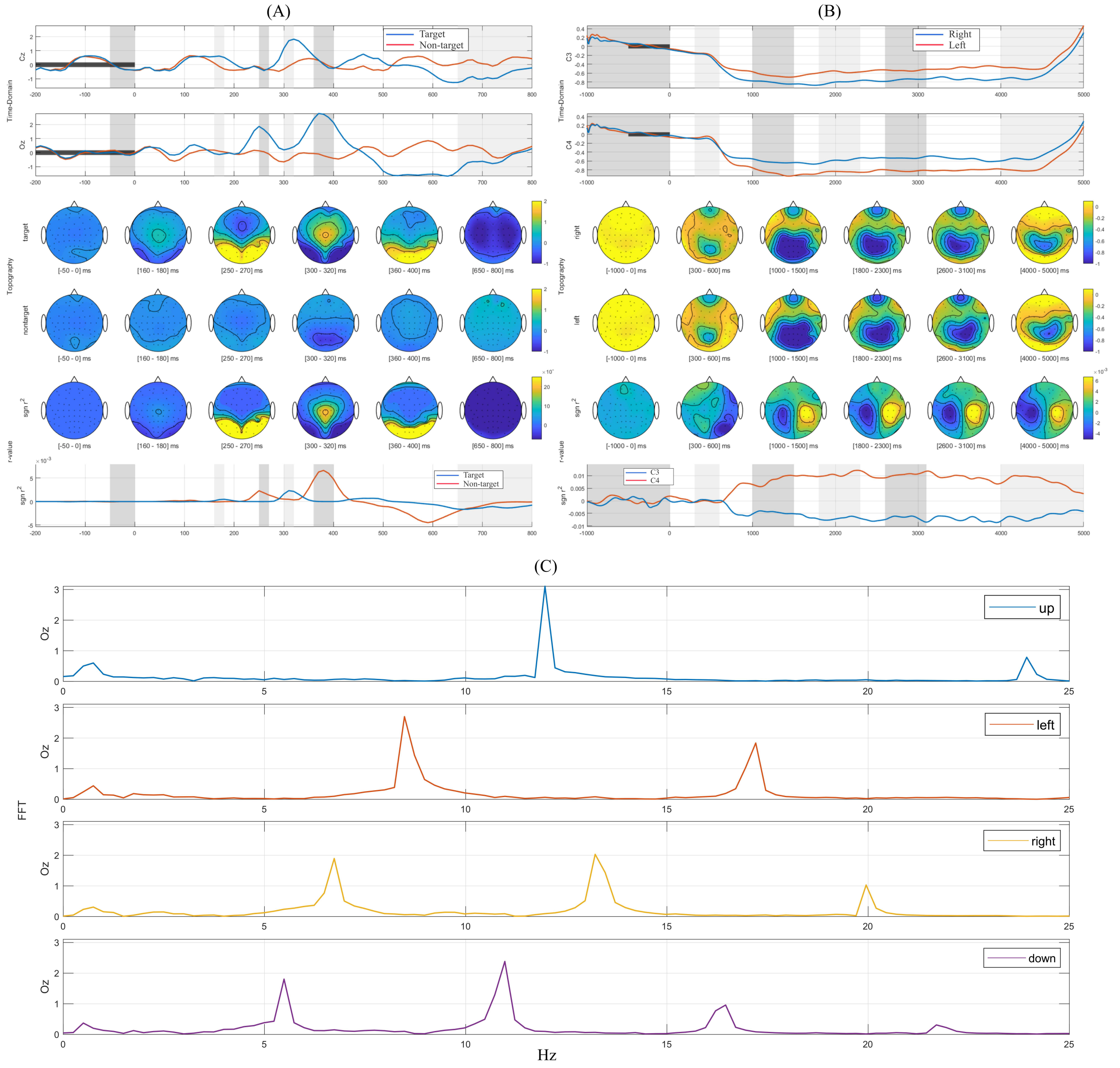

Figure 3. Visualization of P300 responses (A), ERD/ERS patterns (B), and power spectral density (PSD) (C) for ERP, MI, and SSVEP data, respectively. In the visualization of ERP (A) and MI (B) data, first two rows show grid plots in time (x-axis) and amplitude (y-axis) domains for grand-averaged brain responses in certain channels (ERP: Cz and Oz, MI: C3 and C4). The next two rows indicate the topographies of entire brain area for each class corresponding to the certain time intervals which are displayed as gray areas in the grid plot. Fifth and sixth rows present topographic and grid plot, respectively, for signed *r*-values (significance level) between the binary classes. In the visualization of SSVEP data (C), one dimensional data at Oz electrode was extracted and PSD was calculated in frequency range of 0.1 to 25 hz (x-axis).

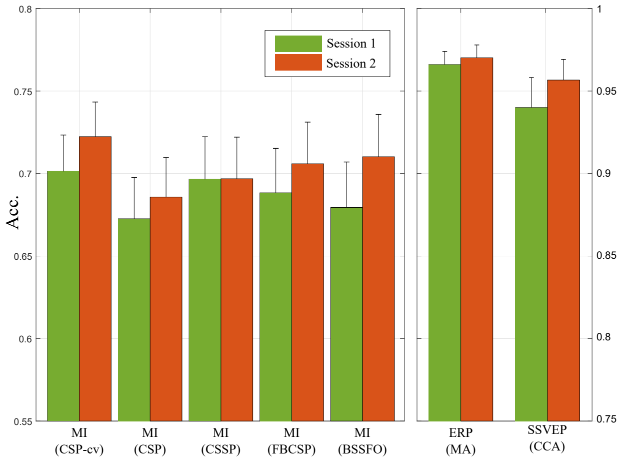

Figure 4. Average decoding accuracies in three BCI datasets over all subjects and sessions. The MI data was validated based on the CSP-cv, CSP and more advanced algorithms (i.e., CSSP, FBCSP, and BSSFO). The decoding accuracies of ERP and SSVEP data were validated based on mean amplitude of ERP features and CCA, respectively.

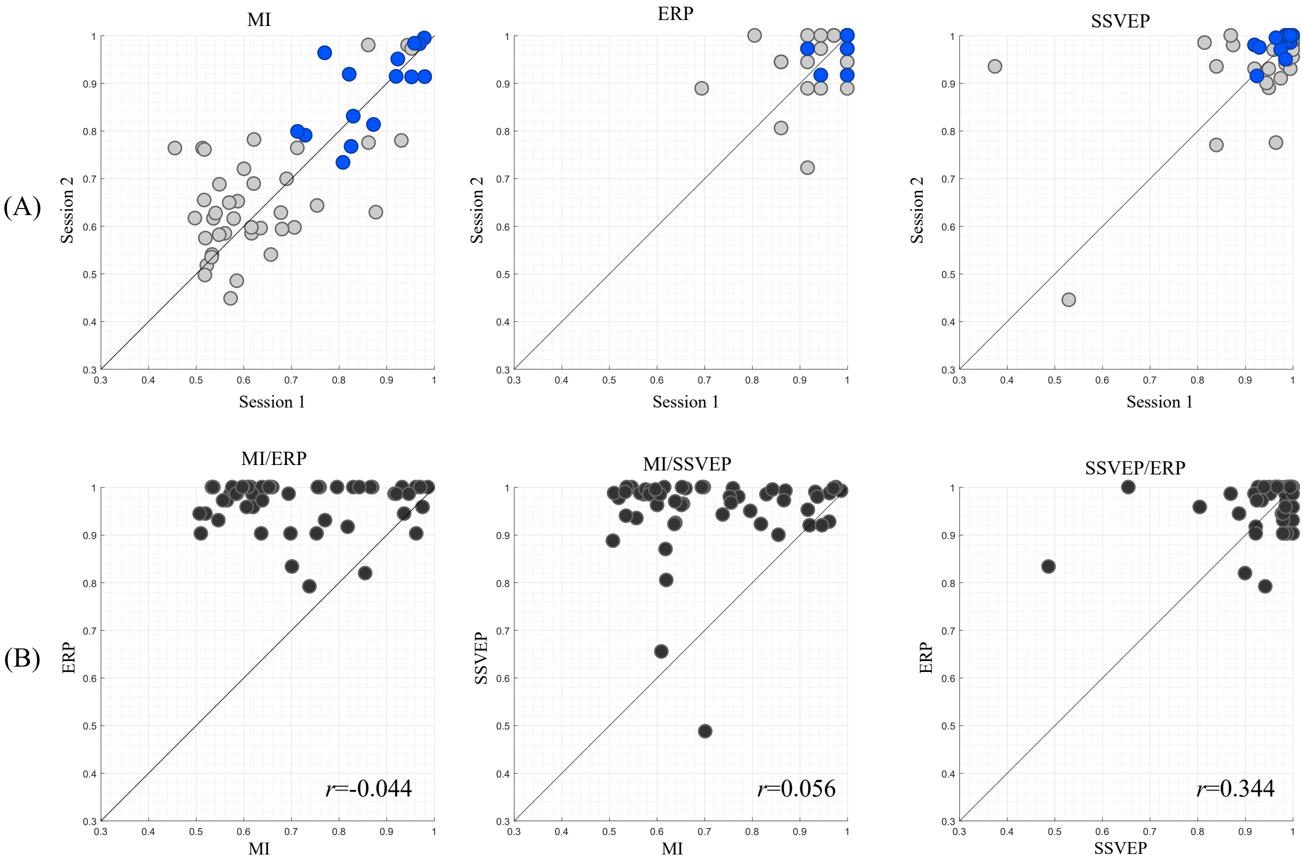

Figure 5. Scatter plots of performance variation across all subjects between sessions and paradigms. The first row shows variations of decoding accuracy in individual paradigms between sessions. Blue and gray circles indicate universally and partially literate BCI users, respectively, calculated in common decoding accuracy for the three BCI paradigms. The second row displays performance comparisons between paradigms (r, correlation coefficient).

Manuscript Click here to download Manuscript main.tex

```
This is pdfTeX, Version 3.14159265-2.6-1.40.16 (TeX Live 2015/W32TeX)
(preloaded format=pdflatex 2016.4.6)  13 MAY 2018 23:33
entering extended mode
 restricted \write18 enabled.
 %&-line parsing enabled.
**./main.tex
(./main.tex
LaTeX2e <2016/03/31>
Babel <3.9q> and hyphenation patterns for 81 language(s) loaded.

! LaTeX Error: File `oup-contemporary.cls' not found.

Type X to quit or <RETURN> to proceed,
or enter new name. (Default extension: cls)

Enter file name:
! Emergency stop.
<read *>

l.11 ^^M

*** (cannot \read from terminal in nonstop modes)


Here is how much of TeX's memory you used:
 10 strings out of 493027
 217 string characters out of 6137679
 53546 words of memory out of 5000000
 3650 multiletter control sequences out of 15000+600000
 3640 words of font info for 14 fonts, out of 8000000 for 9000
 1141 hyphenation exceptions out of 8191
 10i,0n,7p,88b,8s stack positions out of 5000i,500n,10000p,200000b,80000s
!  ==> Fatal error occurred, no output PDF file produced!
```

Figure

Click here to download Figure Figure1_channel.eps

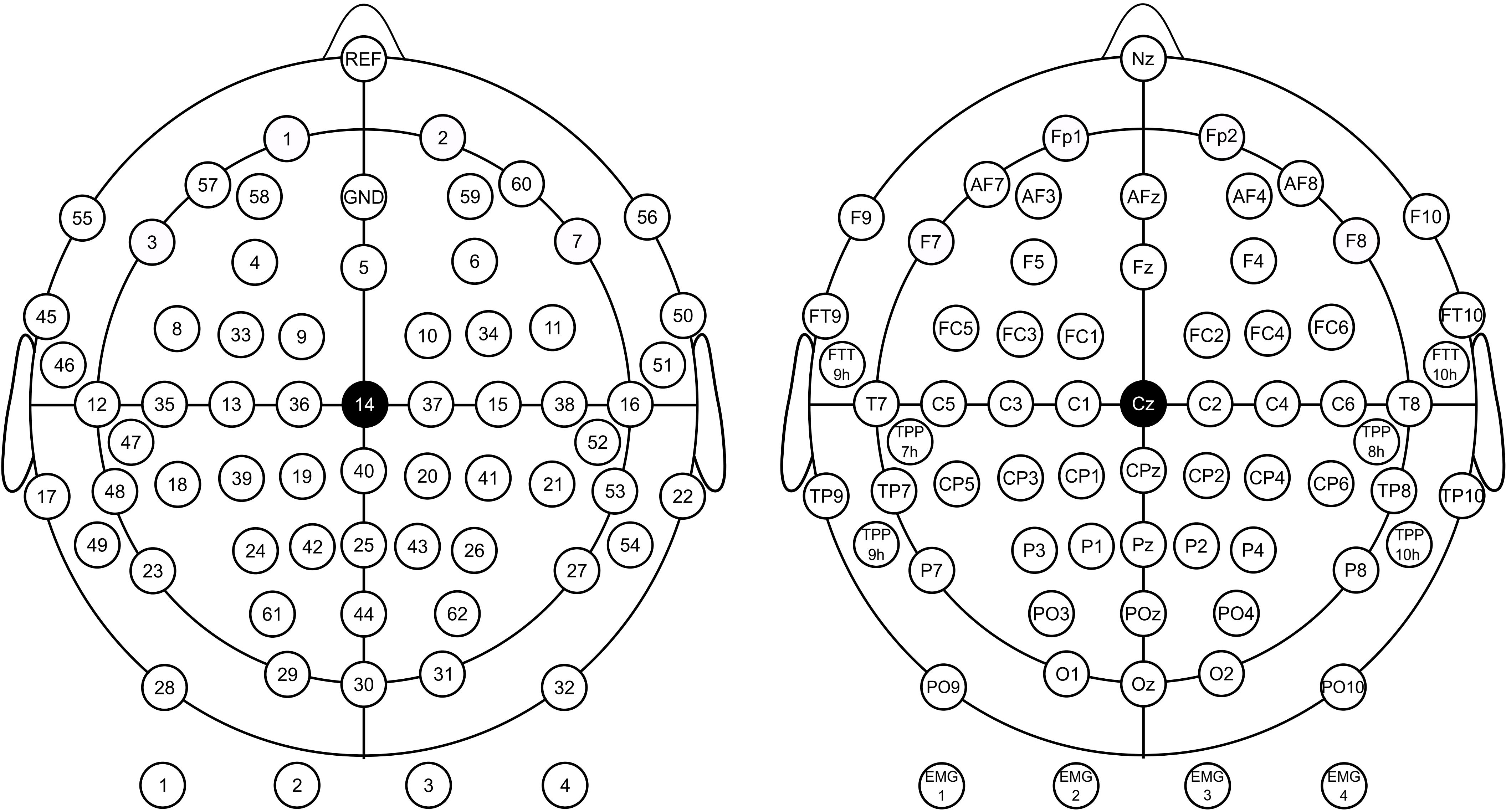

Figure

Click here to download Figure Figure2_paradigm.eps

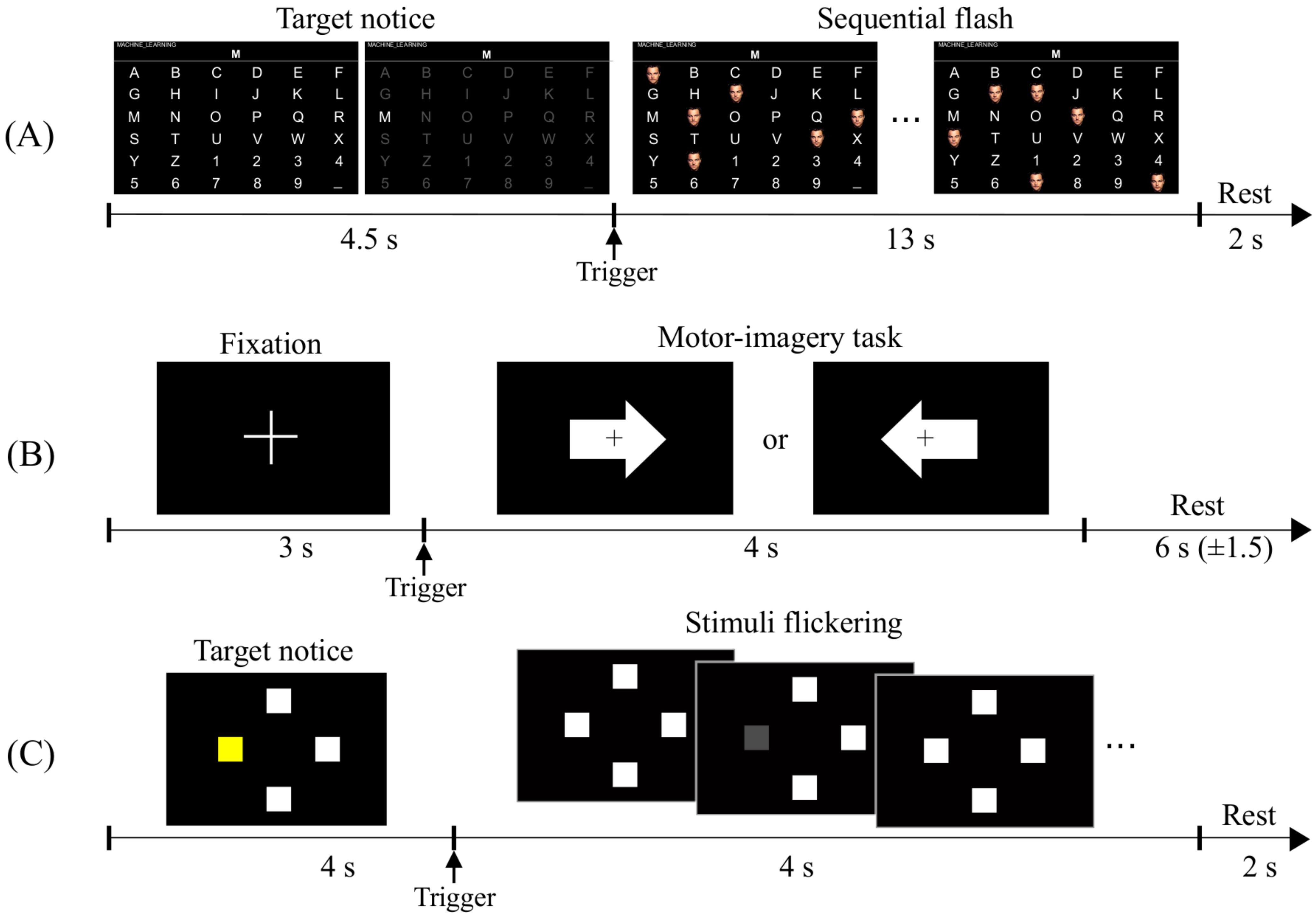

Figure

Click here to download Figure Figure3_visualization.eps

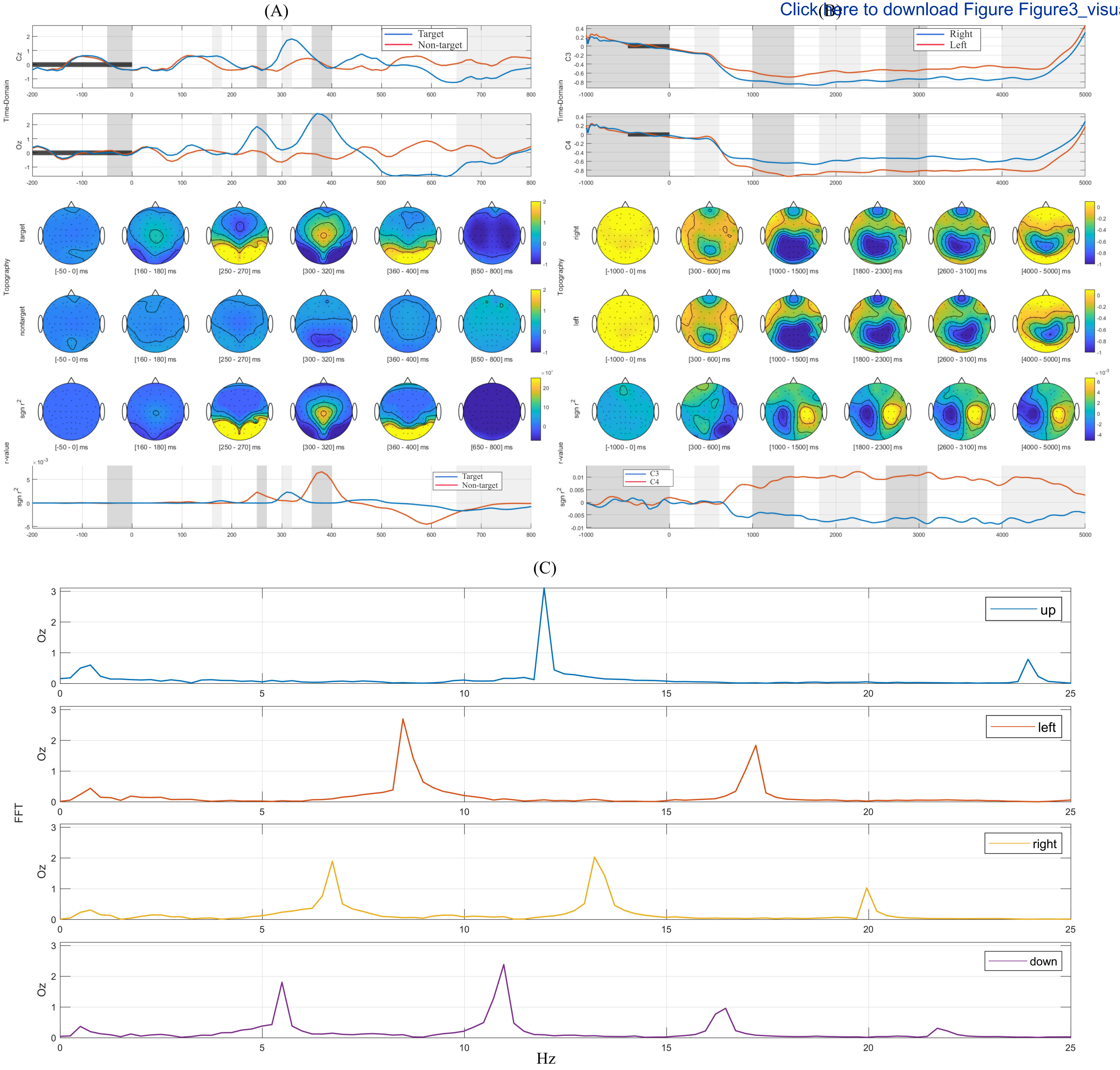

Figure

Click here to download Figure Figure4_bar.eps

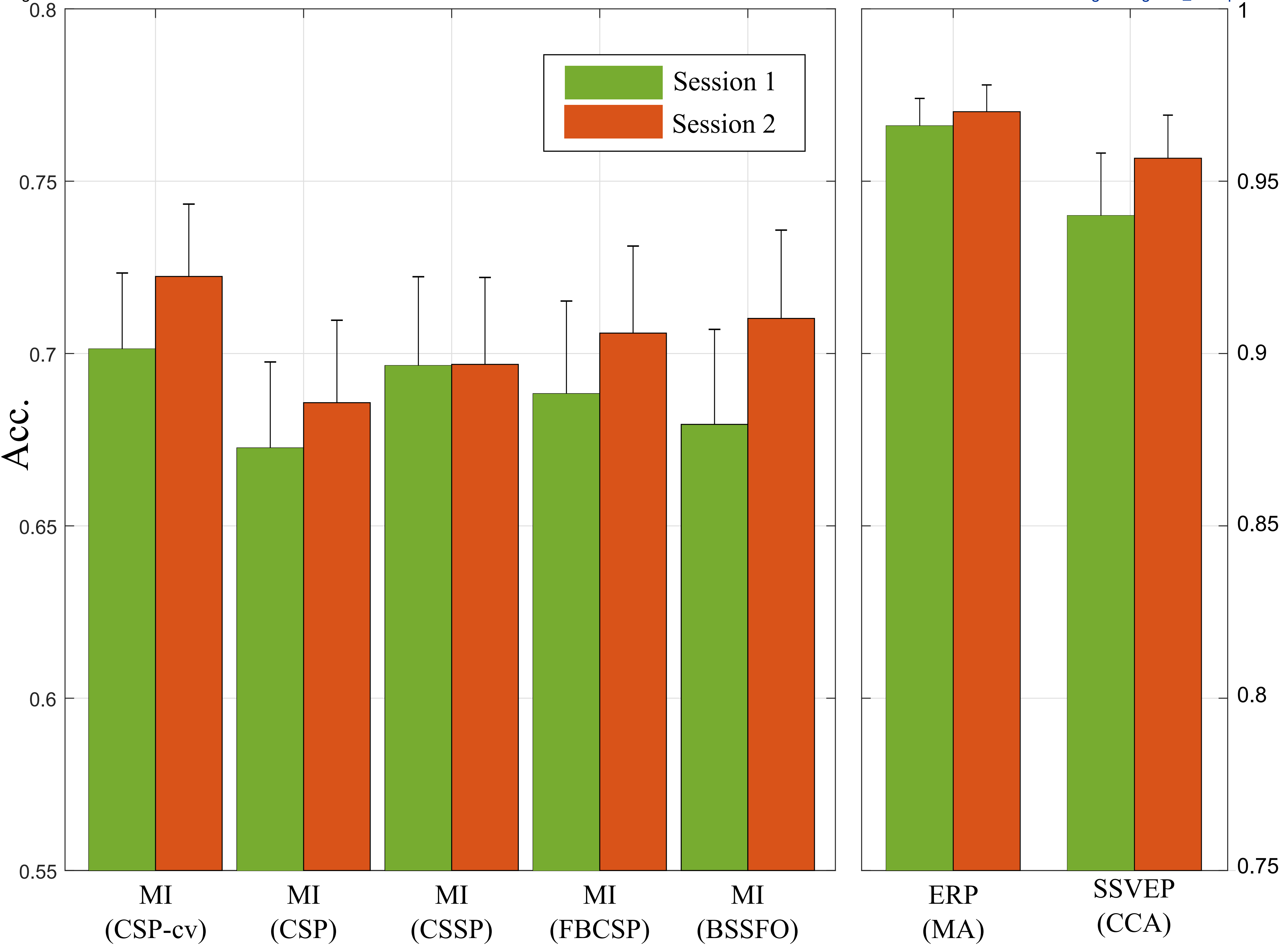

Figure
Click here to download Figure Figure5_scatter.eps

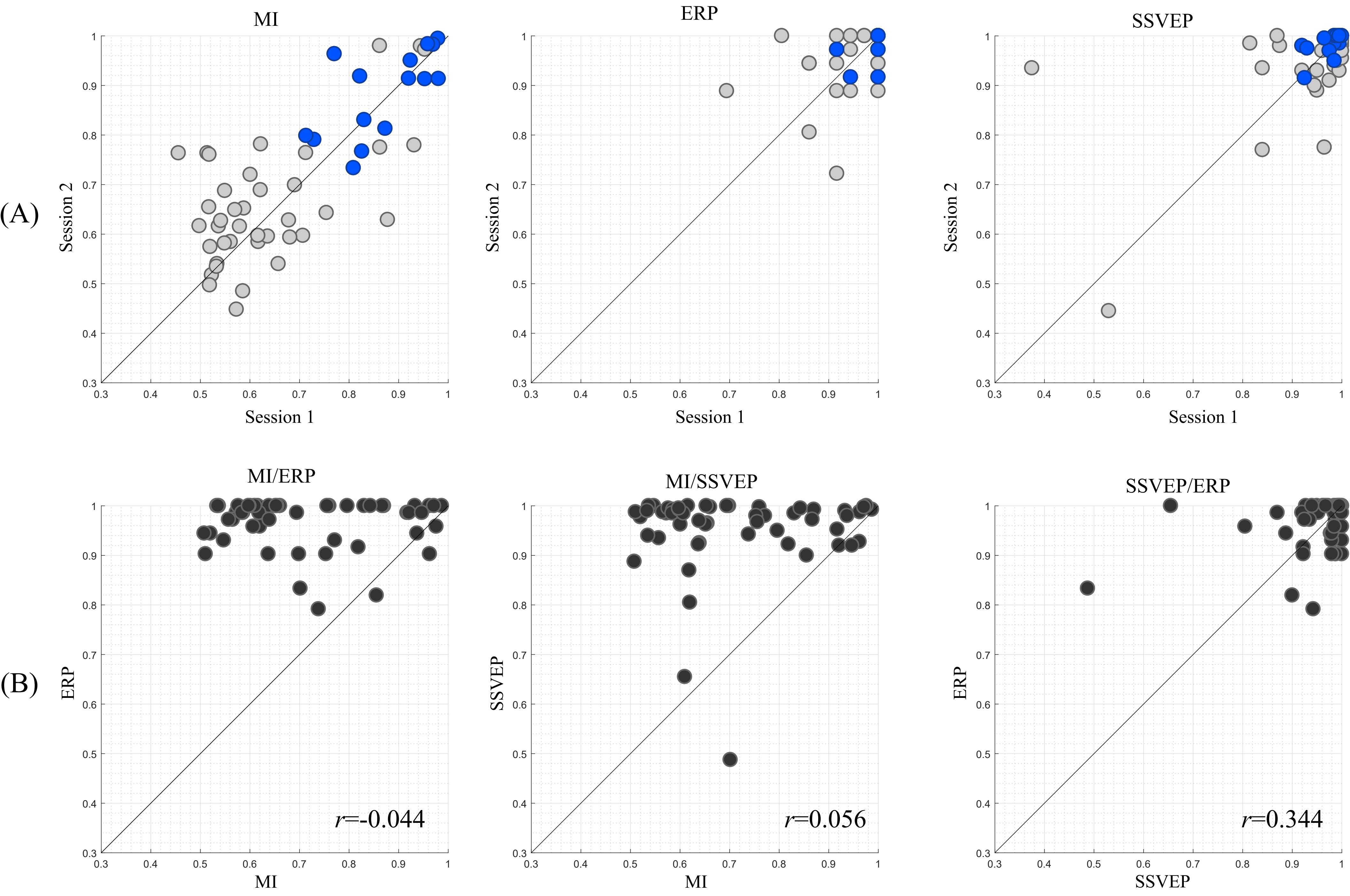

Supplement: giga-d-18-00170_original_submission.pdf [file giz002_giga-d-18-00170_original_submission.pdf]
