# Supplementary material for: EEG dataset and OpenBMI toolbox for three BCI paradigms: an investigation into BCI illiteracy
Source: Gigascience. 2019 Jan 30;8(5):giz002. doi: 10.1093/gigascience/giz002 (PMC6501944; doi:10.1093/gigascience/giz002)
Supplement: response_to_reviewer_comments_original_submission.pdf [file giz002_response_to_reviewer_comments_original_submission.pdf]

Author's Response To Reviewer Comments

Close

Dear editor,

The revision file includes tables and figures which cannot be included in this form.
Please see the attached document for answers to all reviewers' comments.

Kind regards

Close
