# Supplementary material for: EEG dataset and OpenBMI toolbox for three BCI paradigms: an investigation into BCI illiteracy
Source: Gigascience. 2019 Jan 30;8(5):giz002. doi: 10.1093/gigascience/giz002 (PMC6501944; doi:10.1093/gigascience/giz002)
Supplement: response_to_reviewer_comments_revision_1.pdf [file giz002_response_to_reviewer_comments_revision_1.pdf]

Author's Response To Reviewer Comments

Close

Dear editor,

Please see the attached document for answers to all reviewers' comments.
The revision file is uploaded as 'supplementary material'.
Thanks.

Kind regards,
Seong-Whan Lee

Close
