# Supplementary material for: EEG dataset and OpenBMI toolbox for three BCI paradigms: an investigation into BCI illiteracy
Source: Gigascience. 2019 Jan 30;8(5):giz002. doi: 10.1093/gigascience/giz002 (PMC6501944; doi:10.1093/gigascience/giz002)
Supplement: response_to_reviewer_comments_revision_2.pdf [file giz002_response_to_reviewer_comments_revision_2.pdf]

Author's Response To Reviewer Comments

Close

Dear Editor,

Fist of all, thanks for your help. Also, we apologize this delayed process.

I updated all the editor's minor comments in the manuscript.

Please see the file 'Reference_PDF'.

I had one difficulty in putting ORCID ID due to some of the error in latex. I appended all author's ID as below:

%mhlee: 0000-0002-5730-1715
%oykwon: 0000-0001-5498-0540
%yjkim: 0000-0003-3038-4087
%hkkim: 0000-0002-1786-2729
%yelee: 0000-0003-2610-7028
%jw: 0000-0001-7883-9816
%sf: 0000-0003-3397-0647
%swlee: 0000-0002-6249-4996

Thanks.

Close
