# Supplementary material for: EEG dataset and OpenBMI toolbox for three BCI paradigms: an investigation into BCI illiteracy
Source: Gigascience. 2019 Jan 30;8(5):giz002. doi: 10.1093/gigascience/giz002 (PMC6501944; doi:10.1093/gigascience/giz002)
Supplement: reviewer_1_report_original_submission -- Xiaorong Gao — 7/26/2018 Reviewed [file giz002_reviewer_1_report_original_submission.pdf]

Reviewer Report

Title: EEG Dataset and OpenBMI Toolbox for Three BCI Paradigms: An Investigation into BCI Illiteracy

Version: Original Submission **Date:** 7/26/2018

Reviewer name: Xiaorong Gao

Reviewer Comments to Author:

In this paper, the authors built a BCI dataset that included three major BCI paradigms. This study is a welcome addition to the literature, but I have several issues with the manuscript in its current form.

1. This paper is for open access BCI dataset. Authors should provide a download link for the data and codes in the paper.
2. The same authors have published two papers for introducing the OpenBMI toolbox. The authors should explain what are the main differences and similarity among this study and the two previous papers.
3. Recently, several SSVEP-based BCIs studies [7] (Chen et al., 2015; Nakanishi et al., 2018) have been published which proposed some considerable increase in the number of targets. Furthermore, a large number of targets makes sense in some particular situations such as a practical speller application. However, this paper only built 4-target SSVEP-based BCI dataset, which may limit the application of this dataset to some extent. Why not try a large number of targets?
Chen X, Wang Y, Nakanishi M, Gao X, Jung T P and Gao S 2015a High-speed spelling with a noninvasive brain-computer interface Proc. Natl. Acad. Sci. U S A. 112(44): E6058-E6067.
Nakanishi M, Wang Y, Chen X, Wang Y T, Gao X and Jung T P2018 Enhancing detection of SSVEPs for a high-speed brain speller using task-related component analysis IEEE Trans. Biomed. Eng., 65(1): 104-112.
4. Since experiments include offline, training and testing sessions, it means that it will takes a lot of time for calibration, which may lead to high workload for subjects.
5. Provide some more specs about the distance of the view to the screen and the (resulting) visual angle of the stimuli and the whole matrix area.
6. Please provide the mental task of subjects for the MI paradigm.
7. Authors mentioned "the classiÂ¬fication accuracy and information transfer rates (ITRs) were calculated in each sequence." Why not provide the ITR results? Was the gaze-shifting time included in the ITR calculation?
8. Authors should use statistical analysis to investigate whether there are significant differences among different methods.
9. How did the authors determine who was BCI illiteracy? It is better to provide a table to list who is the BCI illiteracy.
10. According to Figure 4, the Session2 results of almost three BCI paradigms were better than the Session1, please explain.
11. The technical validations, which presented in the current study, were relatively few. It is better to provide more analytical results of the dataset.

12. Authors should provide the detailed description of the data (*.mat format). Furthermore, OpenBMI toolbox did not include the Load_MAT function.

Methods

Are the methods appropriate to the aims of the study, are they well described, and are necessary controls included? Choose an item.

Conclusions

Are the conclusions adequately supported by the data shown? Choose an item.

Reporting Standards

Does the manuscript adhere to the journal's guidelines on [minimum standards of reporting?] Choose an item.

Choose an item.

Statistics

Are you able to assess all statistics in the manuscript, including the appropriateness of statistical tests used? Choose an item.

Quality of Written English

Please indicate the quality of language in the manuscript: Choose an item.

Declaration of Competing Interests

Please complete a declaration of competing interests, considering the following questions:

- Have you in the past five years received reimbursements, fees, funding, or salary from an organisation that may in any way gain or lose financially from the publication of this manuscript, either now or in the future?
- Do you hold any stocks or shares in an organisation that may in any way gain or lose financially from the publication of this manuscript, either now or in the future?
- Do you hold or are you currently applying for any patents relating to the content of the manuscript?
- Have you received reimbursements, fees, funding, or salary from an organization that holds or has applied for patents relating to the content of the manuscript?
- Do you have any other financial competing interests?
- Do you have any non-financial competing interests in relation to this paper?

If you can answer no to all of the above, write 'I declare that I have no competing interests' below. If your reply is yes to any, please give details below.

I declare that I have no competing interests

I agree to the open peer review policy of the journal. I understand that my name will be included on my report to the authors and, if the manuscript is accepted for publication, my named report including any attachments I upload will be posted on the website along with the authors' responses. I agree for my report to be made available under an Open Access Creative Commons CC-BY license (http://creativecommons.org/licenses/by/4.0/). I understand that any comments which I do not wish to be included in my named report can be included as confidential comments to the editors, which will not be published.

I agree to the open peer review policy of the journal

To further support our reviewers, we have joined with Publons, where you can gain additional credit to further highlight your hard work (see: https://publons.com/journal/530/gigascience). On publication of this paper, your review will be automatically added to Publons, you can then choose whether or not to claim your Publons credit. I understand this statement.

Yes Choose an item.
