# Supplementary material for: EEG dataset and OpenBMI toolbox for three BCI paradigms: an investigation into BCI illiteracy
Source: Gigascience. 2019 Jan 30;8(5):giz002. doi: 10.1093/gigascience/giz002 (PMC6501944; doi:10.1093/gigascience/giz002)
Supplement: reviewer_2_report_original_submission -- Dheeraj Rathee — 8/5/2018 Reviewed [file giz002_reviewer_2_report_original_submission.pdf]

Reviewer Report

Title: EEG Dataset and OpenBMI Toolbox for Three BCI Paradigms: An Investigation into BCI Illiteracy

Version: Original Submission **Date:** 8/5/2018

Reviewer name: Dheeraj Rathee

Reviewer Comments to Author:

This manuscript presents an open-access BCI dataset consisting of three different experimental paradigms (i.e. ERP, MI, and SSVEP). EEG and EMG data were recorded with 54 participants in two different sessions. In particular, the MS examined the acquired datasets for investigating BCI illiteracy. In addition, the authors introduce OpenBMI toolbox for general purpose BCI studies. While I feel that the analysis and the results provided in the MS are of high quality, I have various comments that I think should be addressed before publication:

1. The MS needs a thorough proof-reading. There are lots of typos, e.g., Abstract (Page 1, line 36): 'several cases of BCI illiteracy than have been previously reported', Page 1, line 54: 'A brain-computer interfaces (BCI)', Page 2, line 8 (in 2) must be written as 2) in), line 13 (amongst). There are many more apart from these, however, it is difficult to list them all here.
2. Why the ground channel used for EEG data recording is different in section 'EEG data recording' (Page 3) and Figure 1.
3. The Section 'Experimental procedure' is not presented properly with lots of repetition and incoherency. I would suggest rewriting paragraphs 3, 4, and 5 (Page 3) in accordance with Table 3 to make it easy for the readers to follow the procedure.
4. In case of performance validation of MI data, it is worth to mention whether the authors considered all or a subset of the CSP components.
5. In BCI illiteracy section, the authors mentioned 'To investigate the rate of BCI illiteracy, decoding accuracies of session one and two were averaged.' However, in the next paragraph, they provided percentages of BCI illiteracy for both sessions separately.
6. The discussion section can be improvised by including further insights into BCI illiteracy and how the findings of the study add to the current state-of-the-art domain knowledge.
7. There is no or limited metainformation presented regarding the open-access dataset, e.g., the process of accessing the dataset, arrangement of the data files (i.e., size, number, and format), and other related information.
8. The result section consists of mean classification accuracies over the 54 participants. However, if possible, the author should include participant-wise CAs for all BCI paradigms and both sessions. This will allow future studies to perform a better comparison of their findings with the current study.
rent study.

Methods

Are the methods appropriate to the aims of the study, are they well described, and are necessary controls included? Choose an item.

Conclusions

Are the conclusions adequately supported by the data shown? Choose an item.

Reporting Standards

Does the manuscript adhere to the journal's guidelines on minimum standards of reporting? Choose an item.

Choose an item.

Statistics

Are you able to assess all statistics in the manuscript, including the appropriateness of statistical tests used? Choose an item.

Quality of Written English

Please indicate the quality of language in the manuscript: Choose an item.

Declaration of Competing Interests

Please complete a declaration of competing interests, considering the following questions:

- Have you in the past five years received reimbursements, fees, funding, or salary from an organisation that may in any way gain or lose financially from the publication of this manuscript, either now or in the future?
- Do you hold any stocks or shares in an organisation that may in any way gain or lose financially from the publication of this manuscript, either now or in the future?
- Do you hold or are you currently applying for any patents relating to the content of the manuscript?
- Have you received reimbursements, fees, funding, or salary from an organization that holds or has applied for patents relating to the content of the manuscript?
- Do you have any other financial competing interests?
- Do you have any non-financial competing interests in relation to this paper?

If you can answer no to all of the above, write 'I declare that I have no competing interests' below. If your reply is yes to any, please give details below.

I declare that I have no competing interests

I agree to the open peer review policy of the journal. I understand that my name will be included on my report to the authors and, if the manuscript is accepted for publication, my named report including any attachments I upload will be posted on the website along with the authors' responses. I agree for my

report to be made available under an Open Access Creative Commons CC-BY license (http://creativecommons.org/licenses/by/4.0/). I understand that any comments which I do not wish to be included in my named report can be included as confidential comments to the editors, which will not be published.

I agree to the open peer review policy of the journal

To further support our reviewers, we have joined with Publons, where you can gain additional credit to further highlight your hard work (see: https://publons.com/journal/530/gigascience). On publication of this paper, your review will be automatically added to Publons, you can then choose whether or not to claim your Publons credit. I understand this statement.

Yes Choose an item.
